# Serum amyloid A – a review

**DOI:** 10.1186/s10020-018-0047-0

**Published:** 2018-08-30

**Authors:** George H. Sack

**Affiliations:** Departments of Biological Chemistry and Medicine, The Johns Hopkins University School of Medicine, 725 N. Wolfe Street, Physiology 615, Baltimore, MD 21205 USA

**Keywords:** Serum amyloid A, SAA, inflammation, amyloidosis, acute phase response (APR), arthritis, apolipoprotein, liver, cytokine, lipopolysaccharide (LPS), myeloid-derived suppressor cells (MDSC), atherosclerosis

## Abstract

Serum amyloid A (SAA) proteins were isolated and named over 50 years ago. They are small (104 amino acids) and have a striking relationship to the acute phase response with serum levels rising as much as 1000-fold in 24 hours. SAA proteins are encoded in a family of closely-related genes and have been remarkably conserved throughout vertebrate evolution. Amino-terminal fragments of SAA can form highly organized, insoluble fibrils that accumulate in “secondary” amyloid disease. Despite their evolutionary preservation and dynamic synthesis pattern SAA proteins have lacked well-defined physiologic roles. However, considering an array of many, often unrelated, reports now permits a more coordinated perspective. Protein studies have elucidated basic SAA structure and fibril formation. Appreciating SAA’s lipophilicity helps relate it to lipid transport and metabolism as well as atherosclerosis. SAA’s function as a cytokine-like protein has become recognized in cell-cell communication as well as feedback in inflammatory, immunologic, neoplastic and protective pathways. SAA likely has a critical role in control and possibly propagation of the primordial acute phase response. Appreciating the many cellular and molecular interactions for SAA suggests possibilities for improved understanding of pathophysiology as well as treatment and disease prevention.

## Background

Homeostasis is essential for most biological systems. Both primordial and adaptable mechanisms exist for reestablishing this important state following perturbations. Among the former, the so-called “acute-phase response” (APR) is prominent. The APR comprises a stereotyped set of physiologic changes (Kushner 1982; Gabay and Kushner 1999; Yoo and Desiderio 2003) that occur as a consequence of inflammation, infection, trauma and other events. The APR involves many physiologic responses – fever, hormonal changes, metabolic alterations. Changes in serum protein levels during the APR are particularly remarkable. Among these, altered serum levels of C-reactive protein (CRP) and serum amyloid A (SAA) are the most notable. Proteins and genes of the SAA family are particularly prominent in the APR. This review emphasizes human SAA genes and proteins but their close relationships to those in murine and other species means that relevant studies from other systems also will be considered when informative.

Both CRP and SAA are present, but at generally quite low levels, in the blood of healthy individuals (SAA is normally found at 20-50 mcg/ml). However, their levels can rise as much as 1000-fold 24 hours after APR onset, largely reflecting *de novo* synthesis in the liver. Consistent with the APR definition, blood levels of both CRP and SAA fall rapidly as the stereotyped APR pattern resolves.

CRP is an endogenous human protein that specifically reacts with the type C polysaccharides of *Streptococcus pneumoniae* (Tillett and Francis 1930*;* Macleod and Avery 1941). Its level also was found to rise after other acute infections. Thus, it has been considered to participate in primordial, endogenous, host-defense against a commonly encountered bacterial pathogen. The serum CRP level is widely used as a non-specific but clinically useful marker for inflammation of many types.

By contrast, the biology of SAA protein(s) has been less clear; both the name and discovery have been more indirect. The term “amyloid” originally referred to material found in plants and presumed to be carbohydrate (*Gk* “amylon” = starch). “Amyloidosis” was used by 19^th^ century pathologists to describe apparently amorphous, infiltrative histopathologic changes in kidney/liver/heart often found in postmortem examinations using light microscopy. Although the term implied that the infiltrating material was carbohydrate, later studies showed that “amyloid” changes comprised protein deposits (Hass 1942) and electron microscopy revealed arrays of microfibrils (Cohen and Calkins 1959). These deposits shared a characteristic birefringence when stained with planar dyes such as Congo red or thioflavin T and this tinctorial property remains the basis for current histopathologic diagnosis. Clinical data implied that the proteins constituting “amyloid” might differ because the deposits were encountered in very different pathologic contexts; many of these have subsequently been defined. For example, amyloid deposits in multiple myeloma comprise fibrillar arrays of fragments of immunoglobulin light chains. Thus, identifying the constituent protein(s) in amyloid deposits is important. In addition to immunoglobulin-derived polypeptides, Benditt and Eriksen (1961) distinguished a protein, referred to as “amyloid of unknown origin” (AUO), isolated by gel electrophoresis in cases of so-called “secondary” amyloidosis (*i.e.* those associated with chronic or recurrent inflammatory conditions). Subsequent studies revealed a distinct amino acid composition, including absence of cysteine and threonine and an amino terminal sequence of R-S-F-F-S (Benditt *et al.* 1971). Levin *et al.* (1972) presented the first complete sequence of a 76 aa protein found in secondary amyloid deposits. The fibril-derived proteins isolated by different laboratories differed slightly in length from 68 – 76 aa but shared common N-terminal residues (Benditt *et al.* 1971; Levin *et al.* 1972; Ein *et al.* 1972a, b). These were referred to as “AA” (*i.e.* “amyloid A”). Antibodies prepared against these proteins identified a small (104 aa) serum protein that was initially presumed and later shown to be their precursor (Linke *et al.* 1975; Meek *et al.* 1986; Prelli *et al.* 1987; Husby and Natvig 1974; Rosenthal *et al.* 1976). Because this was the first non-immunoglobulin serum protein identified as a precursor of amyloid disease deposits, it was named “serum amyloid A” (SAA). Subsequently, it has been shown that SAA is a prominent constituent of APR proteins arising from various stimuli.

**Fig. 1:**
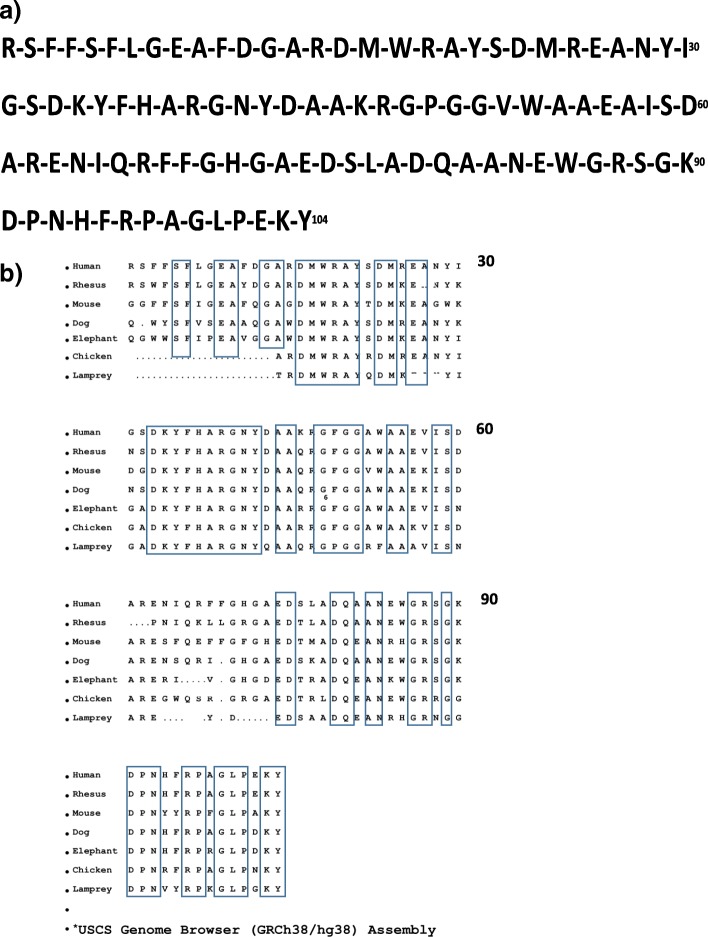
**a** Consensus amino acid sequence for human SAA (SAA1 shown although variants are recognized as well – see Fig. 2) **b** Comparison of SAA sequences in different organisms. Conserved residues noted in boxes. (USCS Genome Browser [GRCh38/hg38] Assembly)

**Fig. 2:**
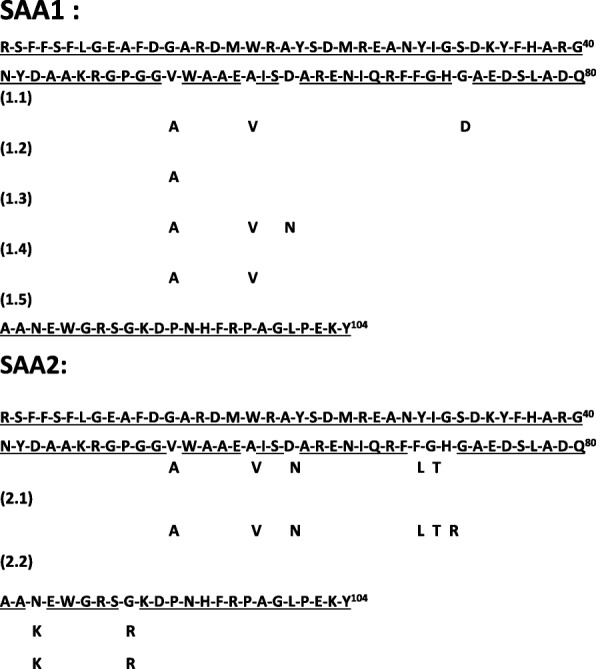
Reported amino acid variants for SAA1 and SAA2 in humans. Underlined regions appear invariant.

The SAA sequence (see Fig. 1a) is remarkably conserved throughout the mammalian radiation as well as in birds and other animals (see Fig. 1b and (Uhlar *et al.* 1994; Jensen *et al.* 1997)). In humans, multiple, apparently minor, variants have been identified. Figure 2 (Faulkes *et al.* 1994; Sipe 1999; Sun and Ye 2016) shows reported sequences. Unfortunately, many past reports did not distinguish among SAA gene and/or protein family members. Unless specified originally, the APR serum species (SAA1 and/or 2) should be considered relevant; other family members, when identified, will be noted in this review.

The APR profile of SAA protein levels in blood is similar to that described for CRP, rising as much as 1000-fold within the initial 24 hours and then returning to very low levels as the event resolves. Although proteolysis of SAA yields the N-terminal fragment(s) found in the microfibrils in secondary amyloid deposits the responsible protease(s) has(ve) not yet been unequivocally identified. SAA is poorly soluble in aqueous solutions; in blood it is partitioned into high density lipoproteins (HDL (Benditt and Eriksen 1977; Benditt *et al.* 1979)). It functions as an apolipoprotein in this regard, possibly changing the character of the HDL particles (see below and (Benditt *et al.* 1979; Hoffman and Benditt 1982; Cabana *et al.* 1989; Kisilevsky and Subrahmanyan 1992)). Aside from its apolipoprotein character, no function was initially identified for SAA although its APR association implied that it might be related to primordial host defense or inflammation, similar to the situation for CRP.

## SAA proteins

Although SAA proteins are small and their sequences and polymorphisms have been carefully catalogued details of their 3-dimensional structure(s) have been elusive. A barrier to fully characterizing these proteins has been their generally poor solubility. The previously recognized tendency of SAA 1, 2 and 4 to associate with HDL lipoproteins (they have been referred to as *apo*lipoproteins) is consistent with this poor aqueous solubility although hydrophobic residues are not strikingly prominent in their primary sequences. Thus, despite their relatively small size, it has been difficult to achieve SAA protein concentrations suitable for NMR study and/or crystallization. Based on earlier algorithms, Turnell *et al.* (1986) proposed a model with two α-helices and a β-sheet region. Stevens (2004) proposed a theoretical structure based on possible homology to the N-terminal domain of arthropod hemocyanins with a predominant helical bundle and a disordered C-terminal region. A soluble fusion protein between SAA and staphylococcal nuclease (Meeker and Sack 1998) showed only α-helical domains by CD. Only a single surface tryptophan residue was identified by iodide quenching.

**Fig. 3:**
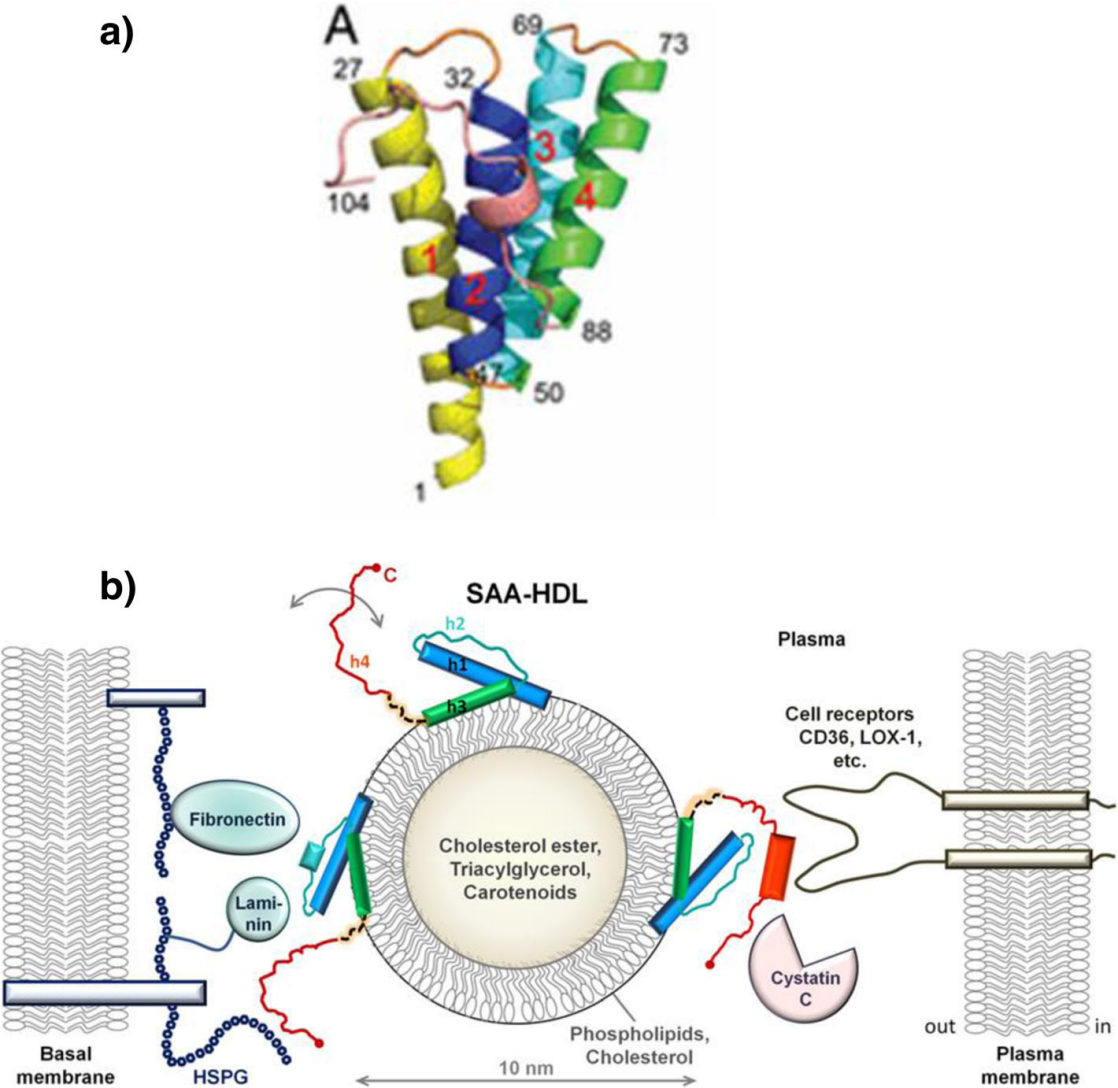
**a** Monomeric three-dimensional structure of SAA1.1 (Lu *et al.* (2014), Copyright [2014], National Academy of Sciences, used by permission). Note 4 α-helices 1 (aa 1-27), 2 (aa 32-47), 3 (aa 50-69), 4 (aa 73-88) and C-terminal tail (aa 89-104). **b** Proposed model for SAA interaction on HDL surface showing potential binding sites to receptors and other molecules. (Frame and Gursky (2016), Copyright John Wiley and Sons, 2016, used by permission)

Lu *et al.* (2014) determined SAA monomer structure by multiwavelength anomalous dispersion to 2.2 Å. As predicted from earlier CD studies (Meeker and Sack 1998), the monomer comprises four α helices in a cone-shaped array (see Fig. 3a). The C-terminal residues are wrapped around the bundle. Further study of His_6_-tagged SAA1 (which could be purified without denaturation) showed a hexamer formed by two identical trimers (consistent with earlier studies in solution (Wang *et al.* 2002)). Interestingly, Wang *et al.* (2005, 2011) earlier had indicated that a less stable octamer resolved to a stable hexamer. Based on this structure, potential sites for binding of both HDL and glycosaminoglycans were predicted to overlap near residues R-1, R-62 and H-71, consistent with a structural basis for competitive binding. This is also consistent with earlier mutation studies (Patel *et al.* 1996) as well as the observation that SAA binding to HDL could be disrupted by heparan sulfate (Noborn *et al.* 2012). The models show that helix h4 contains the site where cleavage usually occurs prior to amyloid fibril formation.

Frame and Gursky (see Fig. 3b) noted that helices 1 and 3 have both hydrophobic and hydrophilic faces. The latter interact with helices 2 and 4 while the hydrophobic domains could bind to the lipid surface of HDL. According to this model, SAA could serve as a “hub” – binding HDL on one side while interactions with glycosaminoglycans, cystatin C, retinoic acid and cell receptors could occur on other domains specifically involving the C-terminus. Binding to each set of domains likely would influence packing of the entire structure due to allosteric changes and the flexible linkage between the N-terminal and C-terminal regions which is most prominent between h3 and h4 helices.

A model for murine SAA3 (where the N-terminal helix has a different primary sequence) presented by Derebe *et al.* (2014) resembled the structure in Fig. 3 but had differences and associated as a tetramer. The core of the tetramer was predicted to be the site for retinal binding. There was no evidence for octamer formation (*cf* (Wang *et al.* 2011)).

## SAA fibrils

As noted earlier, electron microscopy showed that amyloid deposits are fibrillary with relatively similar electron microscopic dimensions. (Upon review, the rabbit tissue deposits earlier (Cohen and Calkins 1959) were likely to comprise SAA-derived polypeptides.) X-ray diffraction patterns of dried (Eanes and Glenner 1968) and native (Glenner *et al.* 1971) amyloid fibrils showed reflections consistent with a cross-β fibrillar model as proposed by Pauling and Corey (1951). Later studies of amyloid fibrils derived from various human sources (including AA fibrils) revealed common synchrotron X-ray diffraction patterns with intense meridional reflections from 4.6 to 4.84 Å (Sunde *et al.* 1997). Subsequent studies have confirmed the common feature of β sheets parallel to the fibril axis; the sheets themselves are generally about 10 Å apart (Eisenberg and Jucker 2012; Knowles *et al.* 2014). In their review of amyloid structures Riek and Eisenberg (2016) emphasized that the interacting domain(s) forming intermolecular β sheets and characteristic cross-β structures may be limited to specific domains of the constituent monomers rather than the entire monomer (they did not have data for AA fibrils).

Different SAA isoforms appear to influence fibril formation, Takase *et al.* (2014) described fibril forms from SAA1.1 as “long and curly” while those formed from SAA1.3 were “thin and straight.” Srinivasan *et al.* (2013) showed that SAA1.1 first formed oligomers followed by a lag phase of several days to form fibrils while SAA2.2 formed fibrils quickly. Interestingly, Mori *et al.* (2014) noted that CE/J mice with a Q30L substitution in both SAA1 and SAA2 are resistant to amyloidosis, presumably implicating a critical domain.

Finding a common unit of β sheet structure has suggested that at least some fibrils in nature may grow via “seeding” from other fibrils or precursors; this was shown by Yan *et al.* (2007) for AA and apoprotein A-11 fibrils in mice. Lundmark *et al.* (2005) reported that at least some proteins in nature can “seed” AA fibril formation in mice. Westermark *et al.* (2010) observed similarities to prion transmission and emphasized nucleation as central to the rate of fibril formation. Such a mechanism could underlie apparent transmission of AA amyloidosis in captive animals (Ranlov 1967).

As noted above, initial sequencing revealed a polypeptide of ≈76 aa common to most AA fibrils (Benditt *et al.* 1971; Levin *et al.* 1972; Ein *et al.* 1972a, b). This sequence has been reported frequently although the N-terminal arginine often is missing. The structure proposed by Lu *et al.* (see Fig. 3a (Lu *et al.* 2014)) features an exposed, non-helical region between aa 69-73, potentially making that and adjacent regions more susceptible to proteolysis. A 1978 study (Lavie *et al.* 1978) showed that human blood monocytes from at least some individuals could degrade SAA into a stable 76 aa species using an outer membrane-associated serine protease; whether this observation can be generalized is unknown. To date, the C-terminal ≈28 aa species has not been detected.

Later studies have found that polypeptides of different lengths can be associated with AA-derived fibrils in different pathologic states. The 76 residue species is commonly found in renal glomeruli. However, Westermark *et al.* (1989) found that about 10% of patients affected with amyloidosis have AA deposits in blood vessels and the renal medulla with relatively little glomerular involvement. Proteinuria was not prominent in these individuals and AA fragments of 45-50 aa were found (all beginning at serine – residue 2). A species of 94 aa was noted in another sample. Thus, while the ≈76 residue fragment is likely the most common constituent of pathologic deposits both longer and shorter species also can be found. Based on accumulating data for other types of amyloid fibrils (see (Riek and Eisenberg 2016)), it is likely that the critical β sheet region for AA fibril formation is relatively short. It is thus possible either that residues at the C-terminal regions are removed *after* fibril formation (and that this process may differ depending on the site(s) of deposition) or that early cleavage of the parent SAA protein leads to monomers differing in length which, themselves, are structurally predisposed to become parts of different types of fibrils. SAA 1 and 2 have isoelectric points ≈5.6 with a more basic N-terminal region and a relative acidic C-terminal domain. Thus, cleavage can alter the pI of the resulting fragment and, possibly, the tissue affinity (Westermark 2005).

In order to form fibrils, SAA cannot be constrained by being bound to HDL. Li *et al.* (2005) showed that transgenic mice overexpressing human heparanase (which led to shortening of heparin sulfate chains) did not have AA fibril deposition in kidney and liver following the established protocol of AEF and AgNO_3_ injection. As noted above (Noborn *et al.* 2012) heparin sulfate disrupted SAA binding to HDL. Digre *et al.* (2016) showed interaction of heparin with both SAA and apoA1 in HDL and that SAA and apoA1 were likely within 25Å on the HDL surface (a distance likely to be too short for the products of heparanase treatment (Westermark 2005) to be effective). These observations are consistent with dependence on both the degree of sulfation as well as the length of the sulfated domains on the effectiveness of fragmented glycosaminoglycans (Takase *et al.* 2016) to release SAA.

The process of SAA fibril formation has an obligate intracellular stage and Jayaraman *et al.* (2017) evaluated SAA’s fate in murine lysosomes. Detailed spectroscopic features were related to environmental pH. In the pH range of 3.5-4.5 SAA1 forms oligomers that are remarkably stable and resistant to proteolysis. This pH-dependent structural transition for lipid-free SAA led to oligomers with 5% α-helix, 50+/- 5% random coil and the remainder β-structure. Such oligomers convert from the α-helix described above (Lu *et al.* 2014) to β-sheets within lysosomes (and, experimentally, other lipid vesicles in which pH can be adjusted) and then disrupt the enclosing membranes and, ultimately, the organelles and cells themselves. Susceptibility of SAA to at least some proteolysis persisted at pH 4.3 (consistent with the possible involvement of cathepsin B (Rὄcken *et al.* 2005)).

Claus *et al.* (2017) used a sensitive FRET assay to confirm that HDL binding helped maintain α-helical conformation and blocked fibril formation. Internalization into lysosomes, freeing SAA from HDL, was essential for fibril development. Furthermore, a sufficient time within the acidic lysosomal environment was required, consistent with relatively slow structural reorganization of the monomer.

**Fig. 4:**
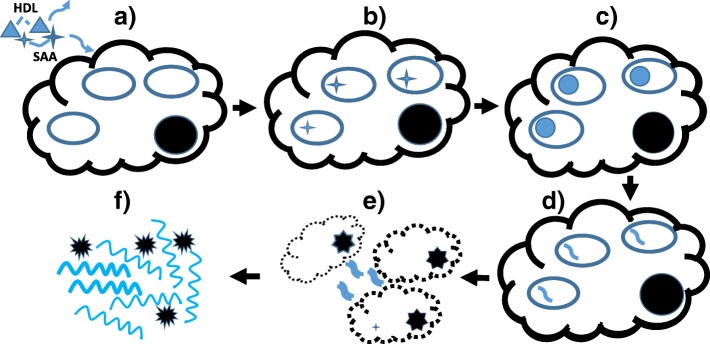
Proposed scheme for SAA fibril formation. **a** HDL-bound SAA dissociates from HDL. **b** {S}AA enters cell via clathrin-coated pits to reside in low pH lysosomal environment. **c** {S}AA monomers undergo structural rearrangement(s) within lysosome. **d** AA oligomers form within cells. **e** Lysis of lysosomal and cellular membranes leads to extracellular oligomers and debris from necrotic cells. **f** AA oligomers extend into fibrils based on β-pleated sheet domain interactions and become visible as tissue “amyloid” deposits with congo red binding. (modified after Claus *et al.* (2017), Copyright John Wiley and Sons, 2017, used by permission). As described in the text, cleavage of SAA occurs during this process, generally yielding a 76 aa N-terminal fragment. Cleavage may involve a serine protease on the cell surface (Lavie *et al.* 1978) but the precise site at which this occurs remains unestablished and the undefined nature of the intracellular species in the early stages (**a-c**) is indicated by {S}AA. By stage (**d**) the AA (post-cleavage) species likely predominates

Figure 4 presents a scheme for AA amyloid fibril formation consistent with these data; it emphasizes the obligate cellular (and intralysosomal) pathway. Beginning with clathrin-mediated endocytosis SAA progresses through lysosomes (with structural reorganization at pH 4.3 with or without cathepsin B proteolysis). SAA oligomers are formed that disrupt membranes leading to cell toxicity with lysis. Finally, extracellular growth of the deposits and pathologically recognizable amyloid fibrils occurs (cellular debris also is present).

While the most prominent constituent of pathologic secondary amyloid fibrils is the SAA-derived “AA” polypeptide other molecules are present as well. Heparan- and dermatan-sulfated glycosaminoglycans and proteoglycans are non-covalently associated (Pepys 2001). In addition, all pathogenic amyloid fibrils contain the plasma glycoprotein serum amyloid P (SAP), a member of the pentraxin protein family (CRP also is a member of this family). SAP contains a calcium-dependent ligand binding site that recognizes an as yet unidentified feature apparently common to all types of amyloid fibrils (Pepys 2001).

## SAA genes and molecular biology

Morrow *et al.* (1981) showed that the level of murine hepatic mRNA for SAA rose 500-fold after inducing the APR by intraperitoneal administration of bacterial lipopolysaccharide (LPS) and that SAA synthesis could rise to comprise 2.5% of total mouse hepatic protein synthesis. This permitted cloning of mRNA for murine SAA by Lowell *et al.* (1986a). All of the genes mapped to murine chromosome 7 (Taylor and Rowe 1984). Two members of this gene family (*Saa1, 2*) show 96% homology over their entire length; a third gene (*Saa3*) is similar in general structure but with distinct sequence differences. In the mouse *Saa1, Saa2 and Saa3* are all acute phase genes. The murine SAA gene family comprises a 42 kb cluster of 5 members (Butler and Whitehead 1996); one (*Saa5*) is a pseudogene.

The striking evolutionary conservation of SAA protein sequences permitted using murine SAA cDNA clones to isolate their human genomic counterparts (Sack 1983). The human SAA gene family is clustered within a 160 kb region of chromosome 11p15.1 (Kluve-Beckerman *et al.* 1986; Sack *et al.* 1989), a region analogous to the chromosome 7 region in the mouse (Taylor and Rowe 1984). Human *Saa1* and *Saa2* are acute phase genes.

**Fig. 5:**
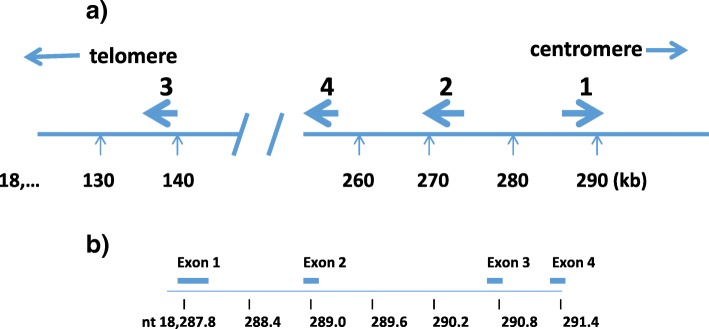
**a** Organization of the four members of the human *Saa* gene family on chromosome 11p. **b** Exon/intron structure of human SAA1 gene (a common pattern for all *Saa* gene family members)

Humans and mice both contain another gene family member (*Saa4*) mapping within the cluster (Steel *et al.* 1993a; DeBeer *et al.* 1996; Uhlar and Whitehead 1999a). The corresponding protein, SAA4, also found as an apoliprotein of HDL, is synthesized constitutively (*i.e*. it is not induced in the APR) and in both murine and human liver and includes an insertion of 8 aa between residues 69 and 70 of *Saa1* and *Saa2*. Figure 5a presents a map of the human *Saa* gene family. All genes share the same organization of 4 exons and 3 introns *(e.g.* Fig. 5b). An 18 aa signal sequence is present in the initial transcript but is removed in the serum proteins.

Despite the remarkable conservation of much of their sequences there is considerable short- and long-range variation among human *Saa* genes and proteins (see Fig. 2), their relationship(s) to the APR and their site(s) of transcription/translation. *Saa1* and *Saa2* are the genes for the classic APR serum proteins in humans and mice. The liver, a major source of APR serum proteins, initially was considered to be the sole site of SAA 1/2 synthesis. However, SAA 1/2 proteins are now known to be synthesized in many other tissues including macrophages, kidney, lung, adipocytes, and mammary gland (Urieli-Shoval *et al.* 1998; Sack *et al.* 2018). Transcription also has been found in synovial cells, brain and mammary gland (Sack and Zink 1992; Tucker and Sack 2001; Larson *et al.* 2003a).

As noted above, the *Saa5* locus is a pseudogene in the mouse. However, the status of the human *Saa3* locus has been confusing. The first reported sequence of a genomic clone (Sack and Talbot 1989) predicted a 104 aa transcribed protein differing from the APR serum proteins due to changes in the N-terminal region. The predicted sequence was similar to that of an SAA-like protein produced by rabbit macrophages following phorbol ester treatment and that functioned as an autocrine inducer of collagenase (Brinckerhoff *et al.* 1989). Later, partial sequencing of a clone from another human library (Kluve-Beckerman *et al.* 1991) found an early stop codon suggesting that this was a non-translated pseudogene. More recently, Larson *et al.* (2003a) detected an RT-PCR product in human mammary epithelial cells stimulated with prolactin or LPS and corresponding to SAA3. Their data predicted an mRNA encoding a protein with an N-terminal sequence matching 29/31 residues of the initial (Sack and Talbot 1989) report. However, their nucleic acid sequence also included an additional T residue at their nt 204 changing the reading frame and, hence, the aa sequence beyond residue 31 with a stop at residue 42 (after the 18 aa signal sequence). In addition, RACE 3’ extension predicted a long 3’ UTR of 406 nt (due to the presumably “early” stop codon generated by the altered reading frame) while the earlier report (Sack and Talbot 1989) predicted only a 145 nt 3’ UTR. Both predicted the same polyadenylation signal (AAUAAAA). The position of the termination codon would lead to a transcript likely susceptible to nonsense-mediated decay (Nagy and Maquat 1998); the corresponding short protein has not been found. GenBank data, based on multiple species, show this “early” stop codon in human, chimp and bonobo genes reflecting a frameshift due to a single “A” nucleotide in the genomic sequence (following codon 30 of the putatively secreted protein (Sack and Talbot 1989)); in these organisms *Saa3* is thus considered to be a pseudogene. In other mammals the sequence is compatible with transcription and translation of a full-length 104 aa protein whose site(s) of translation may be limited to mammary epithelium (Larson *et al.* 2003a) and adipose tissue (Benditt and Meek 1989; Lin *et al.* 2001). Thus, the human *Saa3* locus *is* transcribed but is *not* a source for a 104 aa protein (with presumably the same situation in chimps and bonobos). This distinction is important because *Saa3 is* an authentic gene in other mammals (*e.g*. mice (Benditt and Meek 1989), see also below).

Read-through transcription, presumably due to alternative splicing, between human *Saa* genes has been reported. As shown in Fig. 5, *Saa2, Saa3,* and *Saa4* have the same transcriptional orientation. A low-level transcript connecting *Saa2* (exon 3) with *Saa4* (exon 2) has been identified but the presumptive 208 aa protein has not been found. The distance between these exons is relatively short – 10 kb. By contrast, Tomita *et al.* (2015) reported read-through transcripts connecting exon 3 of *Saa2* with exon 1 of *Saa3* in several human cell lines. The C-terminus of the resulting protein is at aa 42 of the potentially secreted protein, consistent with the frameshift and early stop codon in *Saa3* described above. Such a read-through implies a long distance (≈130 kb) for splicing which appears to be rare (Nacu *et al.* 2011; Hiratsuka *et al.* 2008).

## Control of SAA protein synthesis and serum levels

**Fig. 6:**
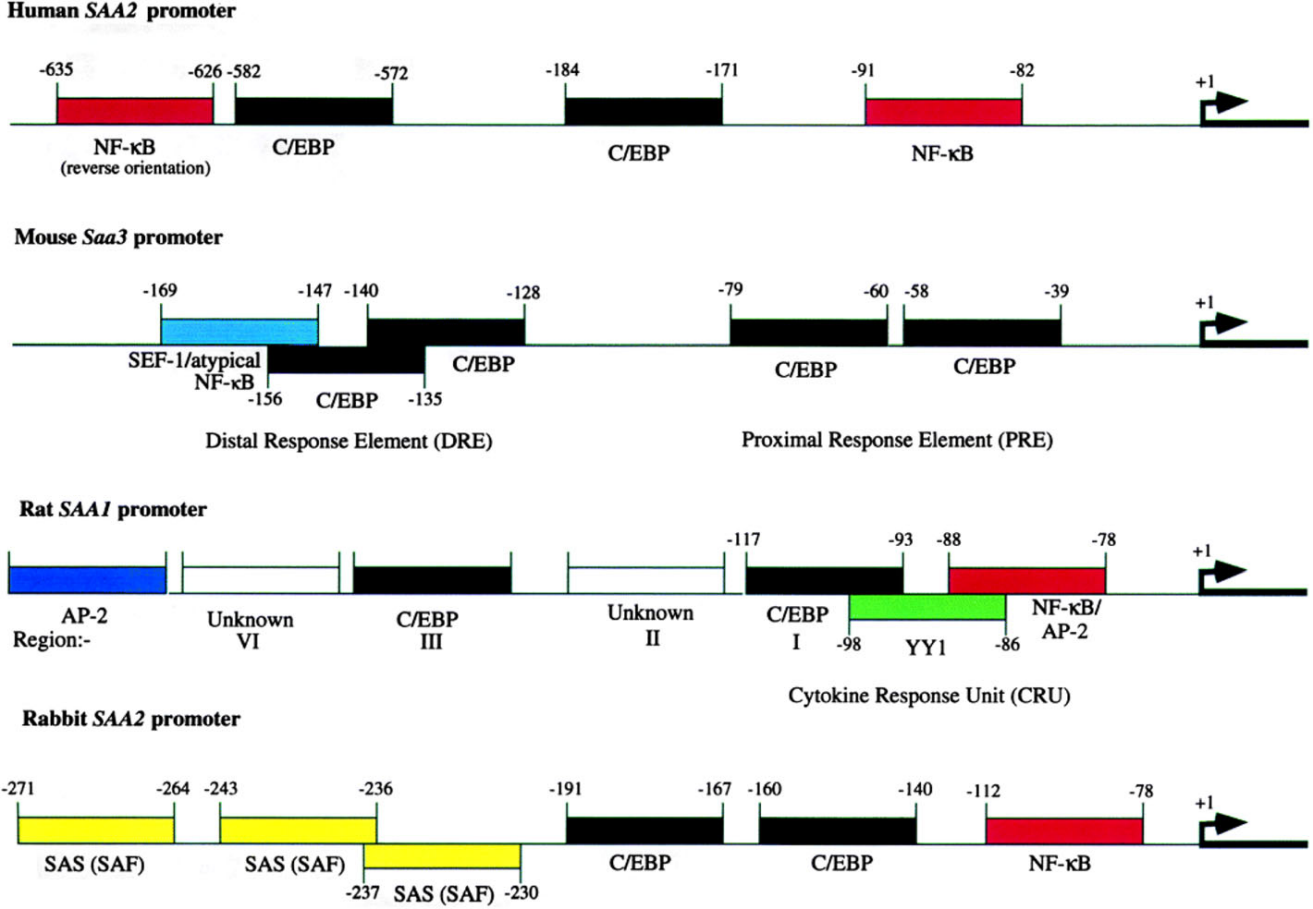
Promoter maps for mammalian SAA genes showing regions for transcription-factor binding. Transcription begins at the arrow (+1). Sites are identified for NF-κB, C/EBP, AP-2, SAS (binds SAF and SEF-1) and YY1. SEF is a factor identified in the mouse distal response element. (Uhlar and Whitehead (1999a), Copyright John Wiley and Sons, 2011, used by permission)

Control of *Saa* gene transcription varies with the individual gene. In addition, details differ in different organisms and different cells and tissues. (This review will emphasize humans and mice although important work also has been done using rat and rabbit systems.) Many studies have emphasized human *Saa1* and *Saa2* genes (those sharing APR expression profiles). As noted above, the APR can be induced with a wide array of stimuli including tumor necrosis factor (TNF), Interleukin-1β (IL-1β), Interleukin-6 (IL-6) and Interferon-γ (IFN-γ) (Edbrooke *et al.* 1989; Betts *et al.* 1993; Edbrooke *et al.* 1991; Woo *et al.* 1987; Edbrooke and Woo 1989). These likely are released from macrophages during the APR. In their earlier review, Uhlar and Whitehead (1999a) described different responses in different cell types and conditions. More recently, de Buck *et al.* (2016) reviewed SAA production in cells of hepatic origin. In some systems, but not all, the temporal order of exposure to stimuli is important. An earlier map of 5’ sites in *Saa* genes (see Fig. 6) indicates some regions incriminated in transcriptional control (Uhlar and Whitehead 1999a; Edbrooke and Woo 1989) but all details have not yet been resolved. IL-1β induces a factor that binds to the NF-κB site, displacing an apparently constitutive repressive nuclear factor that recognizes portions of the adjacent NF-κB and C/EBP sites (Brasier *et al.* 1990).

Vitamin A is required for *Saa* gene expression in mice (see de Buck *et al.* 2016). Derebe *et al.* (2014) showed reduced *Saa1* and *Saa2* transcription in the intestines of mice fed a vitamin A-deficient diet. This was confirmed by real-time qPCR and immunofluorescence. Adding retinol to the epithelial surface of small intestinal explants and of liver after intraperitoneal introduction of retinoic acid led to elevated *Saa1* expression. Using HepG2 cells in the presence of both IL-1β and IL-6 adding retinol or retinoic acid enhanced *Saa1* expression further, consistent with a dietary requirement for vitamin A under normal circumstances. They proposed a model for SAA containing a binding site for retinol (see also below).

IL-6 is most effective early in APR but the combined activity of all factors gives the highest level of transcription (Steel *et al.* 1993b; Uhlar and Whitehead 1999b). Ray & Ray (1996) found 3 homologous pyrimidine-rich octanucleotide sequence motifs (CACCGTCA, CACCCACA, CAGCCCCC) in the -280 - -224 nt region in the mouse that specifically reacted with an “SAA activating factor” (SAF). Mutations in these motifs reduced induction by IL-6 in non-hepatic cells. SAF is a 477 aa zinc-finger protein that becomes phosphorylated during inflammation (Ray *et al.* 2004a) and a mouse transgenic for SAF-1 production developed prominent amyloid deposition (Ray *et al.* 2006). IL-6 is known to bind to gp130 leading to the activation of the STAT3 pathway. Interestingly, although *Saa1* lacks a STAT3 consensus sequence a JAK inhibitor reduced SAA1 transcription by ≈30% but did not eliminate it, thus implicating the STAT pathway in SAA1 transcription (Hagihara *et al.* 2005).

Mice deficient in IL-6 can produce at least a partial APR, depending on the stimulus (Kopf *et al.* 1994). Cytokines other than IL-6 also share the gp130 receptor and may compensate for IL-6 deficiency (Murakami *et al.* 1993). A signaling complex with two gp130 receptors is phosphorylated on an intracellular domain, in turn activating STAT3 and/or MAPK signaling. Hagihara *et al.* (2004) found that an antibody to the IL-6 receptor blocked the APR whereas an IL-1 receptor antagonist or anti-TNF antibody only partially inhibited it. Using a hepatocyte-specific deletion of gp130 in mice, Sander *et al.* (2010) showed that APR protein synthesis was minimized, implicating this receptor and its associated intracellular pathway(s) in both SAA and CRP synthesis. RelA also is essential. Induction of murine APR hepatic *Saa1* was inhibited by RNAi that knocked down STAT3 or RelA, thus incriminating both pathways (Quinton *et al.* 2009, 2012).

Glucocorticoids bind to glucocorticoid receptors that recognize glucocorticoid-responsive elements (GRE). A GRE is found at nt -208- -194 of human *Saa1* but not *Saa2* (Thorn and Whitehead 2002). This difference may explain why adding dexamethasone preferentially stimulates *Saa1* in acute phase stimulation (overcoming a slight bias toward *Saa2* stimulation – for instance in HepG_2_ cells – (Thorn and Whitehead 2003)). Using a reporter assay in human aortic smooth muscle cells Kumon *et al.* (2002) found that SAA1 synthesis responded to dexamethasone but not to other acute phase stimuli. Glucocorticoids bind to their cellular receptor leading to GRE upregulation of IL-6 (Ray *et al.* 1995) and hepatic responsiveness to TNF and IL-1. However, they also can interact with NF-κB subunits in the cytoplasm and increase transcription of IκBα, a cytoplasmic inhibitor of NF-κB. Hence glucocorticoids ultimately inhibit cytokine effects and dampen the APR (Ray *et al.* 1995; Scheinman *et al.* 1995a, b; Auphan *et al.* 1995; Kerppola *et al.* 1993; Knudsen *et al.* 1987).

Two chromosomal regions affecting SAA blood levels were identified in a genome-wide association study (Marzi *et al.* 2010). The first region, 11p15.5-p13, contains the human SAA gene family and the general transcription factor 2 H1, consistent with the map location of the structural gene sequences. The second region implicated (1p31) contains the leptin receptor gene, possibly implicating leptin with APR proteins and SAA biology.

A single nucleotide polymorphism (SNP) in the 5’ flanking region of the human *Saa1* gene (-13 C/T) affects transcription with the -13T allele having greater activity (Moriguchi *et al.* 2005). These workers suggested that this SNP may affect susceptibility to secondary amyloid disease but this has not been confirmed in clinical studies.

Despite impressive levels of SAA1 and SAA2 mRNA seen in APR cells *in vitro* (*e.g*. human Hep3B), translation, although clearly increased, is not proportionally higher (Jiang *et al.* 1995a). In addition, the peak rate of SAA mRNA translation can be 10-fold lower than the rate of mRNA synthesis (Steel *et al.* 1993b). Thus, post-transcriptional regulation likely contributes to blood levels of APR SAA1 and SAA2 proteins. These features are in contrast to those of human *Saa4* where transcription and translation are constitutive. As noted above, *Saa3* is transcribed in murine adipocytes (Benditt and Meek 1989) but less prominently in murine liver. It is also transcribed in rabbit macrophages and other inflammatory cells. As noted earlier, human *Saa3* is transcribed, although apparently not translated, in mammary epithelial cells. In bone, *Saa1* is expressed while both *Saa1* and *Saa2* are expressed in osteoblast-like cells (Koracevic *et al.* 2008).

The steady-state level of SAA mRNA is not only regulated by transcription. As shown earlier (Lowell *et al.* 1986b), mRNA stabilization also is important in maintaining the level. Rienhoff & Groudine (1988) implicated sequences 3’ to the transcription initiation region in determining the half-life of murine SAA3 mRNA. Jiang *et al.* (1995a, b) reported that SAA mRNA levels in Hep3B cells following IL-6 or IL-1β stimulation could be higher than transcriptional rates suggested.

At least some factors controlling SAA mRNA in human and murine cells include: defined binding sites for C/EBP and NF-κB, STAT3 octanucleotide sites responsive to IL-6 and the -13 C/T SNP. Implicating leptin in recent mapping studies widens the scope of APR control and, likely, interactions. Human mammary gland epithelial cells transcribe SAA3 mRNA in response to prolactin or LPS (Larson *et al.* 2003a). However, as discussed above, the mRNA contains an early stop codon and no protein product has been detected.

Less is known about control of murine *Saa3* expression. In mice, a 350 bp promoter fragment can control expression after conditioned medium exposure. The critical region is a 42 bp “distal response element” (DRE) containing 3 functional sections. IL-1, the major cytokine in conditioned medium, is largely responsible for *Saa3* induction but adding IL-6 has a synergistic effect. Integrity of the 3 DRE elements is essential (Huang and Liao 1999). Later studies identified the CCAAT/enhancer-binding protein (C/EBP) and “SAA3 enhancer factor” (SEF). NF-κB and SEF function synergistically at the *Saa3* promoter (Bing *et al.* 2000).

## SAA and APR control (proposed model)

**Fig. 7:**
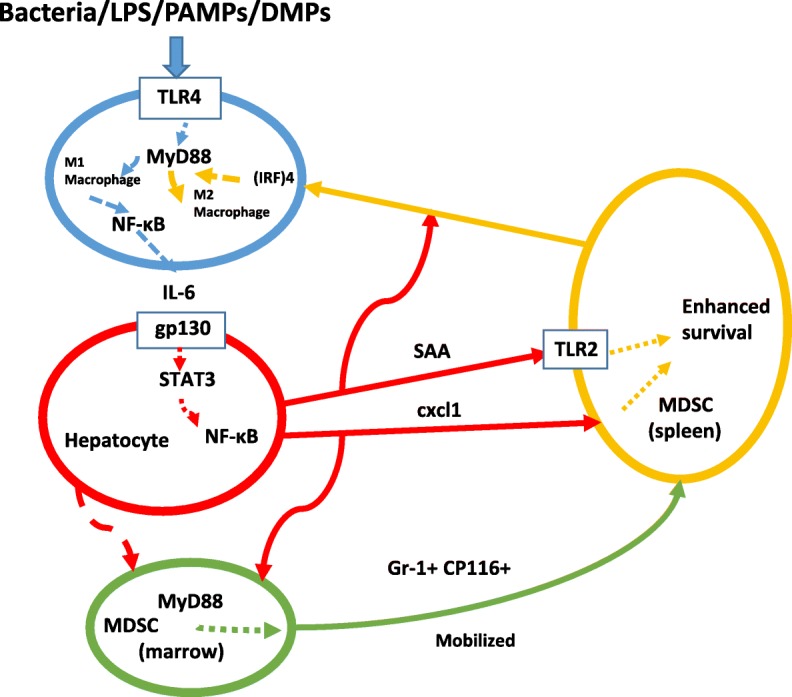
Proposed relationships between cytokines, chemokines and participating cells in APR consistent with current data. Note 3 distinct cell types: 1) macrophages, 2) hepatocytes, 3) myeloid-derived suppressor cells [MDSC]. MDSC can be mobilized from bone marrow and undergo further differentiation in spleen and elsewhere

The ≈1000-fold elevations of serum APR protein levels after 24-36 hours and their subsequent rapid decline imply impressive regulation and feedback. All constituents of this presumptive “loop” have not been characterized fully but murine model systems have been particularly valuable. Pattern recognition receptors on cell surfaces (PAMPS – for bacterial sepsis and DAMPS - damage-associated molecular patterns (Lamkanfi and Dixit 2014)) can be recognized by Toll-like receptors on innate immune cells such as macrophages (of the proinflammatory or M1 phenotype) leading to NF-κB activation, T-cell priming and inflammatory cytokine secretion. Absence of residential T cells can be lethal in such situations (Kim *et al.* 2007). In particular, cytokine IL-6 stimulates hepatocytes via gp130/STAT3 to secrete APR proteins – SAA and CRP. (cxcl1, also known as KC, also is stimulated but less strikingly.) Rapid parallel mobilization of myeloid-derived suppressor cells (MDSC – GR-1+/ CD11b+) through MyD88 also is prominent in the mouse (Sander *et al.* 2010; Delano *et al.* 2007). MDSC are susceptible to apoptosis induction by TNF but in mice their survival (in spleen) is enhanced by both SAA and KC (a chemoattractant for MDSC and granulocytes (Delano *et al.* 2007; Newton and Dixit 2012)). These mobilized MDSC can then mediate feedback inhibition of proinflammatory (M1) macrophage features as well as conversion to pro-resolving (historically referred to as M2) features (more frequently seen in later stages of inflammation, associated with resolution of inflammation and tissue repair) and dampen the entire APR. Sun *et al.* (2015) showed that the conversion of macrophages from proinflammatory (M1) to proresolving (M2) phenotype requires MyD88 and interferon regulatory factor (IRF) 4 in addition to SAA. (Fig. 7 presents a hypothetical cycle including at least some participants).

This model has been tested in mice with a hepatocyte-specific gp130 deletion (*gp103Δhepa*) in which SAA and KC production are not stimulated. In these mice, APR due to CLP (cecal ligation and puncture) protocol for polymicrobial sepsis or LPS can be ≈90% lethal. Normal mice subjected to the CLP showed only ≈25% lethality by the third day. In normal mice, intraperitoneal injection of murine monoclonal antibodies against aa 33-43 of SAA (a well-conserved region – see Figs. 1 and 2) led to 90% lethality at the same time point. Treating the gp130 deletion mice with recombinant SAA1 substantially prevented the enhanced lethality due to CLP (survival was restored to that of normal mice when KC was added as well (Linke *et al.* 2017)). CRP was not effective.

As summarized by de Buck *et al.* (2016) SAA itself can induce a wide array of both cytokines and chemokines. Interrelationships between these have not yet been clarified. Nevertheless, SAA and possibly other APR constituents (not all identified but including chemokine CXCL1 {called KC in the mouse}) participate in a counterregulatory scheme for acute inflammation, critically enhancing its control and down-regulation by prolonging survival of at least one form of MDSC. Other type(s) of MDSC otherwise are susceptible to apoptosis induction by TNF (and possibly other cytokines (Newton and Dixit 2012; Veglia *et al.* 2018)). A feedback loop is thus established for interaction(s) of cells and soluble molecules as APR mediators. This complex process has been rigorously conserved in evolution. Absent or defective function of any of the cellular or molecular participants can lead to prolongation of the APR inflammatory response and likely lethality. Linke *et al.* (2017) proposed that SAA might be therapeutically useful in managing sepsis and other APR-like events.

As described above, chronic inflammation (even at relatively low intensity) can be associated with persistently elevated SAA levels and, ultimately, “secondary” amyloidosis (*e.g*. (McGlennen *et al.* 1986; Chae *et al.* 2009; Lane *et al.* 2017; Kuroda *et al.* 2017)). Participation of SAA in this innate host defense system is consistent with its evolutionary stability. Additional interactions and likely functions for SAA can thus be viewed from this important perspective.

## SAA pathophysiology

While the proposed essential role of SAA in APR regulation (presented above) is compatible with many aspects of its biology, the nature of and basis for other SAA function(s) have remained poorly defined. As noted above, SAA proteins were isolated and described based on their association with the APR and their accumulation in amyloid deposits of “secondary” amyloid disease but no specific function was initially suggested based on these features. The rather small size of SAA proteins makes enzymatic activity unlikely (although this has not been rigorously evaluated). Several observations offer a perspective on possible function(s). Pathophysiologic implications for processes occurring later in life could have developed based on SAA protein chemistry and receptor interactions. Differences in structure and transcriptional patterns of different SAA gene family members suggest a wide range of possible activities.

## SAA binding and receptor(s) (see Table 1)

SAA1 binds to the outer membrane protein A (OmpA, identified by MALDI-ToF) of *E. coli* and, presumably, its homologues in other Gram-negative bacteria (Hari-Dass *et al.* 2005). An *E. coli* strain specifically lacking OmpA showed no SAA binding. Shah *et al.* (2006) found that SAA1 can function as an opsonin increasing uptake of these bacteria by PMNs. Gram-positive organisms (*e.g. Streptococcus pneumoniae* and *Staphylococcus aureus*) did not bind SAA. (Recall that CRP *does* bind to *Streptococcus pneumoniae* (Tillett and Francis 1930; Macleod and Avery 1941).) As noted earlier (Derebe *et al.* 2014) SAA1 can bind retinol and can provide it during an infection – mice deficient in both SAA1 and SAA2 have increased bacterial numbers in spleen and liver following acute infection. Eckhardt *et al.* (2010) found that SAA1/2 derived from mouse colonic epithelial cells (where it apparently was produced constitutively) reduced *E. coli* growth in cultures. These observations are consistent with the idea of SAA being part of a “primordial” host defense and innate recognition system, particularly for Gram-negative bacteria (*cf* CRP as cited above).

**Table 1:** SAA receptor interactions

Receptor	Function	References
SR-B1	Cholesterol efflux, clears SAA	Tomita *et al.* (2015); He *et al.* (2003, 2006); Ye and Sun (2017); van der Westhuyzen *et al.* (2005); Cai *et al.* (2005)
FPRL1	Stimulates MMP-9	Badolato *et al.* (1994, 1995); Gao and Murphy (1993); Fiore *et al.* (1994); Su *et al.* (1999); Patel *et al.* (1998); Furlaneto and Campa (2000); Dufton *et al.* (2010); Ji *et al.* (2015)
FPR2	Phagocyte migration; antiapoptotic	de Buck *et al.* (2016); Gao and Murphy (1993); Furlaneto and Campa (2000); Ye and Sun (2017); Chen *et al.* (2014)
TLR2	Induce IRZF7, IRF4-jmjd3; M2 markers (IL-33, IL-10, IL-1m)	de Buck *et al.* (2016); Ray *et al.* (2006); He *et al.* (2006, 2007); Cheng *et al.* (2008); Ji *et al.* (2015); Ye and Sun (2017); Rosenthal *et al.* (1976); Chen and Moller (2010)
TLR4	Activate NF-κB, AP-1→NOS	Hiratsuka *et al.* (2008); de Buck *et al.* (2016); Sandri *et al.* (2008); Deguchi *et al.* (2013); Li *et al.* (2015); Ye and Sun (2017); Poltorak *et al.* (1998)
RAGE	Activate NF-κB, induce cytokines	Li *et al.* (2015); Yan *et al.* (2000); Rὄcken *et al.* (2003); Kennel *et al.* (2016); Ye and Sun (2017)
Bacteria	Opsonize Gram negative species	Hari-Dass *et al.* (2005); Shah *et al.* (2006); Ye and Sun (2017)

It is important to note that *E. coli* and other Gram-negative bacteria are themselves capable of generating “amyloid” fibrils, known as “curli” (van Gerven *et al.* 2009). These β-sheet containing fibrils are synthesized through a specific pathway involving intracellular assembly, chaperoning and ultimately secretion to the extracellular surface where they become part of biofilms that can protect the organisms. Although biofilms themselves can contribute to bacterial stability and survival it is notable that curli fibrils themselves can be a pathogen-associated molecular pattern (PAMP) and interact with TLR2 receptors on macrophages to induce inflammatory responses (*e.g*. *Nos2* and IL-8 expression) (Tükel *et al.* 2009). Curli-associated pathophysiology appears important *in vivo* (especially via biofilm constituents) where PAMP recognition can lead to an inflammatory response (see Fig. 7). This curli pathway differs from the “opsin” model described above for host protection by SAA. It is likely, but not yet established, that both approaches can provide host defense against at least some Gram-negative bacteria, possibly at different times and locations (*e.g*. nascent bacterial infection vs established biofilms). Whether SAA is directly associated with curli fibril production is unknown.

In addition to opsonin activity noted above SAA physiology including possible role(s) in mediating inflammation and its relation to lipids (details below) implies specific cell surface receptor(s). Delineating such interaction(s) has developed slowly (and likely remains incomplete). A series of studies used a recombinant SAA (rhSAA PeproTech). Badolato *et al.* found that this recombinant induced apparent chemotactic activity with migration, adhesion and infiltration of monocytes and PMN cells as well as intracellular calcium mobilization. These effects could be blocked by pretreating the cells with pertussis toxin (Badolato *et al.* 1994, 1995) and they proposed that SAA could interact with a conventional seven-membrane-spanning G-protein-coupled receptor (Gao and Murphy 1993; Fiore *et al.* 1994). Subsequent studies showed that rhSAA could interact with a homolog of the phagocyte receptor for the bacterial chemotactic polypeptide *N-*formyl-methionyl-leucyl-phenylalanine (fMLP). This homolog (FPRL1) could interact with high levels of fMLP to mobilize calcium, bind with the eicosanoid lipoxin A4 (LXA4) with high affinity and increase arachidonic acid production. Su *et al.* (1999) distinguished rhSAA binding to FPRL1 (rather than FPR) and showed that this interaction could induce calcium mobilization and migration in HEK cells transgenic for FPRL1. rhSAA also bound to human phagocytes and FPRL1-transfected 293 cells at concentrations achievable during inflammation.

SAA-induced cytokine release was shown by Patel *et al.* (1998). Furlaneto and Campa (2000) exposed human blood neutrophils to recombinant human SAA and found 75-400 fold release of cytokines TNF-α, IL-1β and IL-8. This effect was not seen when either normal or acute phase HDL (from febrile patients) was used, suggesting an effect of lipid-free SAA. He *et al.* (2003) confirmed this and showed that overexpression of FPRL1 (also called FPRL1/LXA4R, LXA_4_ and ALX) in HeLa cells increased NF-κB and IL-8 luciferase reporter gene expression after SAA exposure and that proximal events in the pathway included calcium mobilization, MAPkinase (ERK1/2 and p38), and NF-κB activation. The effect also was blocked by pertussis toxin (consistent with earlier observations implicating a G-protein-coupled receptor) as well as an antibody against the N-terminal domain of FPRL1. The SAA effect was significantly reduced in the presence of LXA4 which interacts with the FPRL1 receptor.

In 2007, El Kebir *et al.* (2007) showed that SAA extends the lifespan of PMN cells by suppressing apoptosis – it stimulates phosphorylation of the proapoptotic protein BAD by activating ERK and Akt preventing mitochondrial dysfunction and caspase-3 activation. Importantly, they showed that 15-epi-LXA_4_ triggered by aspirin overrides this effect, suppressing ERK and Akt signals. These observations are consistent with SAA’s prolongation of PMN participation in inflammation and antagonism by 15-epi-LXA_4_. In mice with deletion of Fpr2 (murine FprL1/FPR2 homologue) macrophages and PMNs failed to respond to SAA-mediated chemotaxis (as noted earlier (Su *et al.* 1999; Patel *et al.* 1998)).

Tomita *et al.* (2015) reported binding of human SAA3 (only the short, 42 aa N-terminal species containing the TFLK region – see above) to CHO cells expressing human LDL receptor leading to ERK phosphorylation and MAP kinase signaling. The level of stimulation was similar to that produced by oxidized LDL. Binding of the same SAA3 recombinant to HeLa cells was enhanced by expression of CD36, a phagocyte class B scavenger receptor. Using recombinant SAA (combining SAA1 and SAA2 isotypes) Baranova *et al.* had previously shown that CD36 overexpression in HEK293 cells led to JNK- and ERK1/2- mediated proinflammatory cytokine (IL-8) signaling (Baranova *et al.* 2005, 2010).

O’Hara *et al.* (2004) associated SAA and FMLPL1 gene expression in synovial tissue by noting matrix metalloproteinase (MMP) expression in individuals with inflammatory arthritis (see also below).

Dufton *et al.* (2010) created mice with a deletion of FPRL1 (also called FPR-2) and examined ischemia-reperfusion and carageenan-induced paw edema. Interestingly, phosphorylation of ERK in response to SAA was *not* diminished in the *fpr*^*-/-*^ mice consistent with SAA interaction with other receptors as well.

Relating SAA to lipid transport and cholesterol mobilization also involves receptor interaction(s). This is discussed in the section on lipid biology below. Importantly, Marsche *et al.* (2007) emphasized considering the lipidation status of SAA in determining its cholesterol acceptance. Cholesterol efflux from cells involves the ABCA1 and/or SR-BI pathways.

Toll-like receptors also have been implicated. He *et al.* (2006, 2007; Cheng *et al.* 2008) showed that SAA can induce IL-12, IL-23, G-CSF and granulocytosis. The latter two effects could be inhibited by anti-TLR2 antibody. HeLa cells expressing TLR2 had a good response to SAA that was blocked by Toll/IL-1 receptor/resistance deletion mutants of TLR1, TLR2 and TLR6. *Tlr2*^*-/-*^mouse macrophages had reduced SAA-induced gene expression compared with wild type cells. TLR2 was implicated as a responsive receptor using transgenic HeLa cells expressing TLR2. A solid-phase binding assay showed SAA interaction with the ectodomain of TLR2. Ji *et al.* (2015) noted that T-cell mediated hepatitis was accelerated by SAA1 acting through TLR2. Similar results were not seen in parallel studies using TLR4 (He *et al.* 2007). The SAA effect was reduced using macrophages from tlr2^-/-^ mice.

TLR4 also was implicated in SAA interactions (Sandri *et al.* 2008). Treating murine peritoneal macrophages with SAA induced NO production; this depended upon ERK1/2 and p38 MAPKs. However, this effect was lost when the cells were derived from C3H/HeJ or C57BL/10ScCr (lacking a functional TLR4 receptor). The intracellular pathway appeared to be independent of MyD88. Hiratsuka *et al.* (2008) reported that murine SAA3 bound more strongly to TLR4 in lung endothelial cells and macrophages than to other leucine-rich repeat family members. SAA3 stimulated NF-κB signaling via TLR4, enhancing metastasis. Other studies (Deguchi *et al.* 2013) indicate that murine SAA3 binds TLR4/MD-2 to activate the MyD88-dependent pathway. Li *et al.* (2015) showed that some recombinant human SAA species required TLR4 and RAGE to stimulate expression of double-stranded RNA-activated protein kinase R and release of serum high mobility group 1 box (HMG1) release from murine macrophages.

It is important to note that many studies of SAA have used recombinant protein(s) produced in *E. coli. (e.g.* rhSAA 300-13 and 300-53 PeproTech). Simple aa changes in at least some recombinant molecules may affect their physiology (although they also may aid solubility). Bacterial endotoxin or LPS, which may stimulate TLR4, may be present in such preparations but the manufacturer claims that the level is <0.1ng/mcg SAA protein. The endotoxin level found in amebocyte preparations was even lower (<0.05 ng/mcg SAA protein). Thus, contamination was very low, if not absent, and experiments uniformly controlled for this. (*e.g*. (Sun *et al.* 2015; Badolato *et al.* 1994, 1995)).

The receptor for advanced glycation end products (RAGE) in a soluble form can bind to murine AA amyloid (Yan *et al.* 2000). Heterogeneity in glycation may influence AA fibril formation and/or deposition (Rὄcken *et al.* 2003). A synthetic form of RAGE (VC1) which has a highly basic surface can specifically bind to murine AA deposits (Kennel *et al.* 2016).

These observations support cytokine-like interaction of SAA with several cell-surface receptors. ALX has been studied most thoroughly but TLR2 and TLR4 also appear to be important in at least some aspects of the inflammatory response. Controversy remains regarding induction by SAA which was not shown by Kim *et al.* (2013). Nevertheless, other more recent studies are consistent with interactions which, however, differ with the study system(s) used. Ye and Sun (2017) reviewed SAA/receptor interactions.

## SAA and lipids

Early studies showed SAA association with lipids, particularly HDL (Benditt and Eriksen 1977; Benditt *et al.* 1979). Predicted amphipathic features for SAA monomers implicated regions for lipid binding (Meeker and Sack 1998; Lu *et al.* 2014; Frame and Gursky 2016). Kisilevsky & Manley (2012) estimated that 95% of circulating SAA is in the HDL fraction (consistent with (Benditt *et al.* 1979) showing only 5-6% of SAA reactivity at the top of a density gradient). Using an egg phosphatidyl choline monolayer they also showed high affinity of SAA for phospholipids, similar to that of apo A-1. As noted above, the SAA monomer is poorly soluble in aqueous environments, justifying use of a fusion protein (SAA and Staphylococcal nuclease) for biophysical characterization (Meeker and Sack 1998). Early reports, however, noted that a “small proportion” of SAA can be found in a lipid-free form in human plasma (van der Westhuyzen *et al.* 1986; Coetzee *et al.* 1986). Relationships between SAA, HDL and cholesterol not only vary temporally but also depending upon the environment. In particular, SAA is involved with cholesterol metabolism in both normal physiologic and inflammatory conditions. Interpreting all available studies is complicated by hydrophobic partitioning of SAA monomers.

The physiologic relevance of studies using delipidated SAA has been questioned (Ye and Sun 2017). However, it can be estimated that an equilibrium must exist such that ~50 ng/ml of SAA (presumably monomeric) free of HDL and, possibly, free of lipids, should exist under baseline conditions and that this concentration could rise to ~50 mcg/ml in APR (as noted but not quantified in (van der Westhuyzen *et al.* 1986; Coetzee *et al.* 1986)). Yamada *et al.* (2009) used surface plasmon resonance to estimate binding of human SAA isotypes and HDL. Their data showed K_D_ values for recombinant SAA family members - 1.1, 1.3 and 1.5 - of 1.4 X 10^-5^, 1.8 X 10^-5^, and 3.7 X 10^-6^, respectively. They also estimated the concentration of lipid-free SAA as a fraction of total SAA in patients with rheumatoid arthritis (in whom the serum concentrations were elevated) as 10-40 mcg/ml, consistent with the estimate above. In mice deficient for ApoA-1 *and* ApoE some recombinant SAA was found in a “lipid-poor fraction” of serum as well as in VLDL and LDL fractions although this does not correspond to the *in vivo* situation (Cabana *et al.* 2004). Thus, while lipophilicity of SAA and, particularly, its partitioning into HDL, accounts for ~95% of the protein in serum, a small lipid-free fraction may exist in equilibrium with lipids although details of this fraction remain elusive. Even in quiescent cells in culture a low level of lipid turnover and remodeling is present. This is likely modified qualitatively and quantitatively during the dynamic changes accompanying the APR as well as in other inflammatory conditions. We will consider both acute and chronic conditions. The following studies emphasize the steady state.

Tam *et al.* (2002) loaded murine macrophages with ^3^H cholesterol, cholesterol oleate or oleate alone and exposed them to HDL (50 mcg/ml) from either normal or APR mice. The latter showed reduced oleate to cholesterol ester conversion (*i.e*. reduced ACAT activity) and an increased release of labeled cholesterol (enhanced CEH activity). Trypsinizing HDL (eliminating both apoA-1 and SAA) eliminated this activity with SAA2.1 specifically being essential for this effect. The same effect could be produced using apoprotein-containing liposomes.

Delipidated SAA at 10 mcg/ml promoted cholesterol and phospholipid efflux from HeLa cells, an effect attributed to the ABCA1 transporter (Stonik *et al.* 2004; Qian *et al.* 2017). However, even when the ABCA1 transporter was inhibited (in fixed cells or in fibroblasts from individuals with Tangier disease where the transporter is defective (Rust *et al.* 1999)) reduced efflux was seen. By contrast, similar exposure to apoA-1 could not mediate efflux in cells defective for ABCA1 (Tangier disease or fixed cells), implying that delipidated SAA might use an alternative (undefined) pathway to mediate lipid release. In Chinese hamster ovary (CHO) and Hep G2 cells both delipidated SAA (at both 10 and 30 mcg/ml) and APR murine HDL (containing SAA) increased cholesterol efflux 2-fold (over murine baseline HDL alone) (van der Westhuyzen *et al.* 2005). Using BLT-1 (an inhibitor of scavenger receptor B1 {SR-B1}), intracellular cholesterol release induced by delipidated SAA was reduced (but not eliminated). When added together, SAA and HDL synergistically increased efflux. SAA induced cholesterol efflux in an apparently ABCA1- and SR-B1- (*i.e.* Tangier and BLT-1-treated cells, respectively) independent manner, suggesting 3 efflux pathways.

SAA binding to SR-B1 inhibits HDL binding (Cai *et al.* 2005). It also permits SAA uptake and cleavage within macrophages where degradation – leading to AA fragments - may occur (Kluve-Beckerman *et al.* 2002) (recall Fig. 4).

Labeled delipidated SAA (at 1.25 and 10 mcg/ml) bound to HeLa cells transfected with CLA-1 (human orthologue of SR-B1). Unlabeled SAA and helical polypeptides L-37pA and D-37pA could compete for this binding but an L3D-37pA polypeptide (containing 3 D-amino acid substitutions interrupting the helical structure) did not compete. Confocal microscopy colocalized transferrin and SAA in the endocytic recycling compartment. HDL competed with delipidated SAA for CLA-1 binding. Exposure of CLA-1 expressing HeLa cells to SAA and HDL led to IL-8 secretion as well as activation of MAPKs (He *et al.* 2003).

Treating HEK293 cells stably expressing human ABCA1 with delipidated SAA (10 mcg/ml) led to HDL particles with higher density, larger diameter and slower electrophoretic mobility than those following apoA-1 treatment. Increasing ABCA1 protein by liver retinoic acid receptor (RXR) agonists increased lipid release (Abe-Dohmae *et al.* 2006).

In HEK-293 cells ABCA1 mediated cholesterol release to lipid-free SAA (10 mcg/ml) (He *et al.* 2007) However, overexpressing SR-B1 in HEK-293 cells did not lead to cholesterol release in response to lipid-free or lipid-poor SAA (in contrast to (Abe-Dohmae *et al.* 2006)). These SR-B1 transfected cells transferred cholesterol only to lipid-phosphatidyl choline-associated SAA. By comparison with CHO and HepG2 cells the cell type studied was at least partially responsible for the differences because HEL-293 and HEK-293 cells transfected with SR-B1 showed minimal cholesterol efflux in response to lipid-free and lipid-poor SAA while efflux *was* found in CHO cells and fibroblasts. Thus, both the cell type and SAA lipidation status must be considered.

Hu *et al.* (2008) used the model of LPS-induced APR inflammation in mice to examine intact mouse hepatocytes as well as those with a knockout of the ABCA1 transporter. They concluded that ABCA1 is required to generate HDL within hepatocytes using either SAA or apoA-1. Both delipidated SAA and apoA-1 interacted with ABCA1 causing release of cellular lipids. In ABCA1 knockout mice, no HDL was found in the plasma following LPS stimulus and SAA secretion was reduced (compared with wild-type) and largely found in the low-density fraction. SAA was increased within the liver, however, consistent with the APR pattern. Adding either lipid-free SAA or apo A-1 to fibroblasts derived from both types of mice caused release of cellular lipids from wild-type but not ABCA1-knockout cells. Getz and Reardon (2008) suggested that apoA-1 may mobilize cholesterol and phospholipids from the inner to the outer leaflets of the cell membrane in preparation for exovesiculation to form at least primordial (possibly discoidal) HDL in the presence of amphipathic protein domains. Such nascent HDL particles may require LCAT to convert them to spherical HDL.

Mice with deletions of SAA 1.1 and SAA 2.1 showed no effect on HDL levels in the LPS-induced APR. However, HDL particle size increased due to increases in surface lipid rather than proteins (deBeer *et al.* 2010). The murine SAA3 gene remained intact in these mice, however, and, as reviewed earlier, has considerable sequence conservation especially in the C-terminal region.

Reconciling, at the molecular level, all studies relating SAA to HDL cholesterol is difficult. The studies have used cells from humans, mice and hamsters (in addition different to cell types, *e.g.* CHO, HepG2, fibroblasts, HEK293, HeLa). Furthermore, quantitation of cholesterol flux has been difficult to compare among the systems. An additional complexity is that the precise molecular status of the SAA used in “lipid-free” and “lipid-poor” studies is not clear. In this regard, Kinkley *et al.* (2006) suggested that “lipid-free SAA” may be aggregated even at low levels but this may be part of an equilibrium situation as discussed above. An additional complication (introduced above) has been the relation(s) of the studies to the APR with its attendant cytokine changes (for example, the sequelae of the LPS response) in intact organisms. Ye and Sun (2017) also noted that some recombinant SAA proteins used in reported studies contain aa changes (including N-terminal methionine and D61N and H72R). Moreover, Chen *et al.* (2014) reported differences among SAA isotypes and specific receptors. Specifically, SAA1.1 and SAA2.2 were similar in FPR2 activation and ERK phosphorylation while all tested species gave similar results using a TLR2-dependent luciferase reporter. They also explored the possibility that LPS contamination of recombinant SAA (made in *E. coli*) could affect signaling – using macrophages from Tlr3-deletion mice (Poltorak *et al.* 1998) they found that SAA1.3 induced higher levels of TNFα and IL-1 receptor antagonist transcription. In addition, SAA1.5 promoted the highest IL-10 expression while being less active in IL-1β induction. Thus, the SAA isoform(s) present may differ in receptor selectivity.

Several reasonable summary conclusions can be formulated. 1) The ABCA1 transporter is involved in cholesterol efflux (Stonik *et al.* 2004; Qian *et al.* 2017). 2) SAA promotes an increase in cholesterol ester hydrolase, leading to increased free intracellular cholesterol. 3) SAA reduces activity of macrophage ACAT. 4) HDL containing SAA is targeted to the macrophage within which it can be loaded with free cholesterol (increased by steps #2 and 3) for transport. 5) Although the scavenger receptor SR-B1 may be involved in SAA recognition/uptake it is not the only mediator. 6) The *in vitro* data implicate multiple pathways. 7) Different SAA isoform(s) likely contribute to different downstream activities (*e.g.* Fig. 2) but most studies have not distinguished the species in molecular detail and commercially available SAA proteins often have small aa changes that can assist with their solubility.

Based on the structural data for SAA monomers (Meeker and Sack 1998; Lu *et al.* 2014) and the model proposed by Frame and Gursky (2016) (Fig. 3a, b) the potential for hydrophilic domain exposure after HDL binding has suggested regions responsible for fibronectin and laminin binding (aa 39-41 and 29-33, respectively). Residues toward the C-terminus (including aa 70-104) include regions implicated in binding to heparan sulfate (aa 83-102 (Ancsin and Kisilevsky 1999)), cystatin C (11 86-104 (Spodzieja *et al.* 2013)) and receptors LOX 1 and CD36 (Tomita *et al.* 2015). Such interactions could permit SAA-mediated binding and possibly internalization into cells with these receptors for these species. Other contents of HDL also could be internalized through this mediation (*e.g.* fat-soluble vitamins). These same structural considerations could help explain the C-terminal region’s stimulation of cholesterol ester hydrolase while the N-terminal region reduces ACAT and LCAT (Kisilevsky and Tam 2003; Hosoai *et al.* 1999) thus changing cholesterol from esterified to nonesterified species and increasing transport from cells to HDL.

## SAA and cholesterol salvage

The most prominent changes in serum SAA levels (both 1 and 2, often not distinguished in past reports) occur in the APR as reviewed above and transport and partitioning of SAA under these conditions has been studied extensively. Inflammation, both acute (including the APR) and chronic is central to SAA pathophysiology. Plasma HDL apoproteins change during the APR. Specifically, the fraction of apoA1 in HDL falls while that of SAA rises (Ye and Sun 2017; Kisilevsky and Manley 2012). In addition, HDL_3_ (denser fraction) preferentially associates with SAA and becomes physically altered with an increase in relative size while remaining a polydisperse mixture. The fractional apoprotein composition of HDL (particularly HDL_3_) can shift such that SAA comprises as much as 80% of the total with an SAA:apoA1 ratio of nearly 10:1 (van der Westhuyzen *et al.* 1986). Such changes are consistent with SAA’s lipophilicity as well as its altered synthesis (and relative concentration) during the APR.

Kisilevsky, Manley and coworkers (Kisilevsky and Manley 2012; Kisilevsky and Tam 2003) noted that the evolutionary conservation of SAA and its relation to the APR (reviewed earlier) strongly support a central role for this family of proteins in host survival. They defend the notion that SAA mobilizes cholesterol from macrophages at sites of acute injuries, infections, etc. Under such circumstances cellular debris accumulates in macrophages which then release cytokines that induce the APR (*e.g.* IL-1, IL-6, TNF). Subsequent SAA synthesis and release alter HDL composition as described above as well as the affinity of the reorganized HDL for lipid-laden macrophages. Such a role would be consistent with early survival and evolutionary maintenance. Hosoai *et al.* (1999) emphasized that SAA alone did not lead to reduced HDL cholesterol among the lipoproteins.

Despite this role in “survival” (which, along with APR control, could have been valuable early in life and selected for evolutionarily), it remains important to also consider role(s) of SAA proteins in physiologic processes that, at least in humans, have accompanied longevity as well as more chronic biologic and medical situations that presumably did not initially contribute to evolutionary APR preservation. At least in part, these considerations help explain multiple, often confusing, observations and possibly additional roles for SAA proteins.

## SAA and atherosclerosis

The intimate relationship between cytokines and SAA synthesis and release as well as the SAA/HDL/cholesterol relationships discussed above have implicated SAA proteins in atherosclerosis. Clearly, this is a process complicating longevity and would not necessarily have been related to evolutionary conservation of these proteins and their genes (cf Getz and Reardon 2008; Kisilevsky and Manley 2012). Rather it has developed more recently, largely in older individuals. In this context the notion of “APR” has less relevance and a chronic role is likely more important.

Ross (1993, 1999) reviewed evidence for inflammation as a basic feature of atherosclerosis. His work (as well as that of many others) emphasized important features. Particularly notable are: 1) endothelial dysfunction, 2) chronicity, 3) intimate relationship to serum lipids, particularly LDL, 4) participation of monocytes/macrophages and T-cells (less frequently PMN cells), 5) cytokines, and 6) local tissue remodeling. These processes have been reviewed extensively and will not be presented in detail here.

In addition to their APR changes, both CRP and SAA levels are correlated in chronic inflammatory conditions (Ridker *et al.* 2000; Jousilahti *et al.* 2001). As early as 1994, Meek *et al.* (1994) detected SAA mRNA in atherosclerotic lesions in human cells. O’Brien *et al.* (2005) detected both SAA and lipoproteins in murine vascular disease lesions. Chiba *et al.* (2011) showed that HDL containing SAA bound to proteoglycans from vascular endothelium leading to lipoprotein retention within the vessel wall. Zhang *et al.* (2017) found that SAA could induce a change in the phenotype of vascular smooth muscle cells from a quiescent and contractile form to a synthetic, proliferative form and that the latter could be important in development and propagation of atherosclerosis. Using adenovirus expressing SAA in *Apoe*^*-/-*^ and *Rag*^*-/-*^ mice, Thompson *et al.* (2015) found prominent aortic and brachiocephalic vascular lesions containing high numbers of macrophages. They also noted that this effect was particularly prominent early after SAA induction. Local production of SAA – especially within the lesion(s) was important.

IL-1α induced SAA synthesis in aortic smooth muscle cells (O'Brien and Chait 2006). Kumon *et al.* (1997) distinguished transcriptional regulators of SAA in human aortic smooth muscle cells where C/EBP is prominent (as compared with HepG2 cells). Wilson *et al.* (2008) documented SAA stimulation of vascular proteoglycan synthesis. SAA also stimulated synthesis of phospholipase A_2_, apparently by transcriptional control (Sullivan *et al.* 2010), and the association of SAA with HDL blocked this effect consistent with local direct activity of SAA on lipid metabolism. Wroblewski *et al.* (2011) noted that the combination of SAA and endothelial lipase was associated with reduced HDL cholesterol formation – a situation that could accompany chronic inflammation. Association studies using SAA gene SNPs have suggested relationships to carotid artery intimal media thickness, HDL level and cardiovascular disease (Carty *et al.* 2009) but these have not yet been confirmed in large populations. Interestingly, apoprotein E-deficient mice also deficient in SAA1.1 and SAA2.1 continue to develop atherosclerotic lesions (De Beer *et al.* 2014).

Mice with a hepatocyte-specific knock-out of gp130 (IL-6 signal transducer) and with an atherosclerosis-prone genetic background show less aortic atherosclerosis and decreased macrophages in plaques. Humans with variants in IL6ST (the human gp130 homologue) show significant association with coronary artery disease, particularly involving ostia of coronary arteries (Luchtefeld *et al.* 2007).

**Fig. 8:**
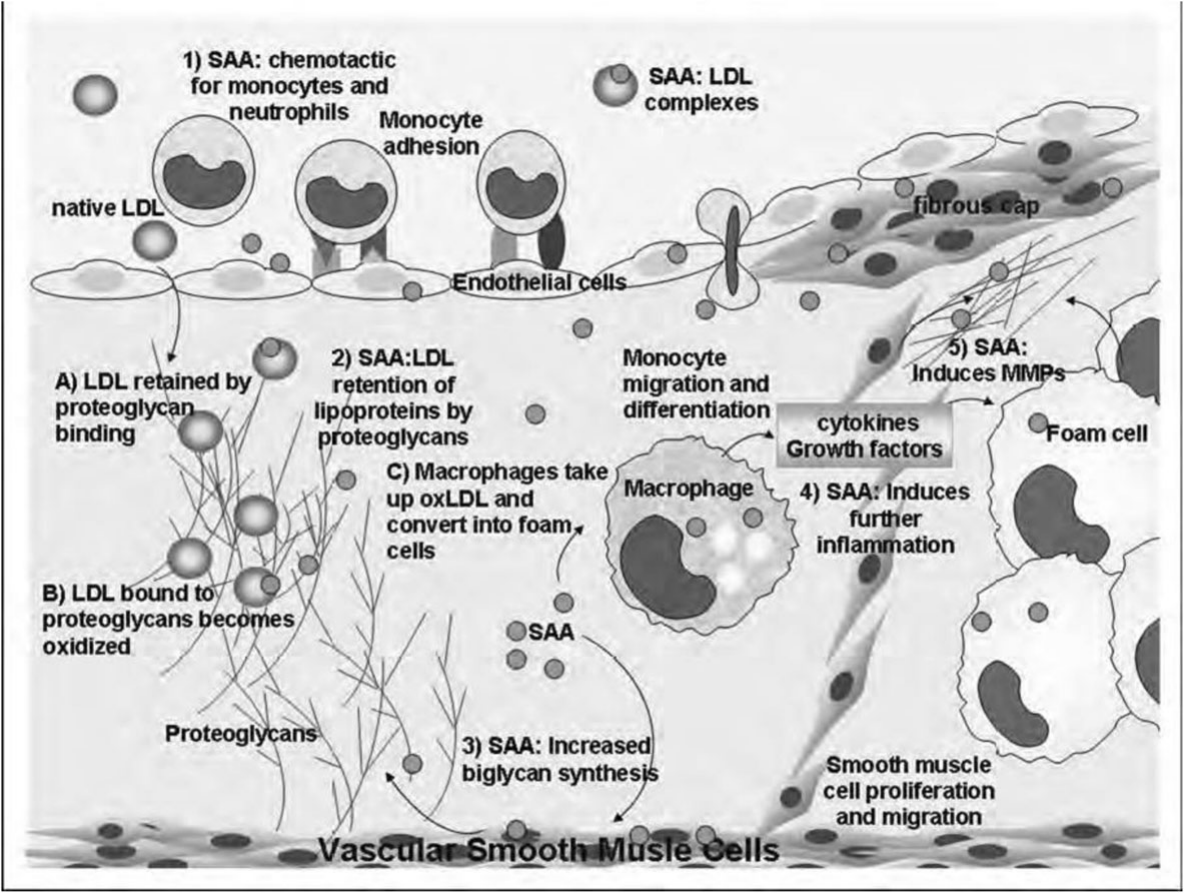
Proposed sites for participation of SAA in atherosclerosis. (King *et al.* (2011), Copyright Lippincott Williams & Wilkins, 2011, used by permission.)

Despite the evidence (above) for APR participation of SAA in cholesterol transport, recent studies have suggested that SAA may impair removal of cholesterol from some peripheral sites. Combined observations (McGillicuddy *et al.* 2009; Annema *et al.* 2010; Feingold and Grunfeld 2010) imply that even in the absence of an inflammatory response (*e.g.* SAA overproduction mediated by a viral vector – see (Meek *et al.* 1994)) SAA can reduce HDL-based cholesterol removal. As reviewed by Getz and Krishack (2016) and King *et al.* (2011) the underlying effect may be modification of HDL structure and/or transport function. The same authors propose the interactions as shown in Fig. 8.

Although there have not been as many direct clinical studies relating SAA and vascular disease, multiple reports have been devoted to the “other” acute phase reactant – CRP, measured by a high sensitivity (hs) assay – as a “biomarker” for atherosclerosis (*e.g*. (Ridker *et al.* 2002)). Historically, CRP levels have been determined more routinely in the clinic than those of SAA so there are large data sets supporting the correlation. Although some reports (*e.g*. (Danesh *et al.* 2004)) have shown that CRP levels are less well-correlated than other common risk factors, the notion of an underlying inflammatory process with associated CRP elevation has appeared valid (Ridker *et al.* 2000; O'Brien and Chait 2006). Reducing cholesterol levels with statin treatment alone is associated with reduced inflammatory marker levels (Ridker *et al.* 2005). Even in asymptomatic individuals with normal levels of LDL but elevated CRP levels statin treatment reduced the CRP level (although the LDL levels fell as well, possibly contributing to the outcome) (Ridker *et al.* 2008). Interestingly, earlier studies (Choi *et al.* 2002; Suissa *et al.* 2006) had reported fewer cardiovascular deaths among patients with rheumatoid arthritis treated with methotrexate and other disease-modifying agents, consistent with the hypothesis that lowering inflammation itself might reduce clinical events. Because IL-1β precedes CRP in the inflammatory cascade (Ridker *et al.* 2017) these and other observations led to the recent CANTOS trial to evaluate the effect(s) of reducing vascular inflammation on cardiovascular events using Canakinumab, a human monoclonal antibody against IL-1β. Including over 10,000 patients with high CRP levels who had had a previous myocardial infarction and were studied over 4 years, this trial showed a significantly lower rate of recurrent cardiovascular events as well as reduced CRP levels (Ridker *et al.* 2017). This important study has provided a “proof-of-concept” for linking inflammation and clinical atherosclerosis (Ibañez and Fuster 2017).

## SAA and cancer

Elevated levels of SAA in blood were detected relatively early in the largely phenomenologic studies of blood protein changes in cancer (Rosenthal and Sullivan 1979). SAA has been evaluated as a possible serum biomarker for many tumors including ovarian (Moshkovskii *et al.* 2005; Edgell *et al.* 2010), lung (Sung *et al.* 2011), renal (Sung *et al.* 2011; Tolson *et al.* 2004; Paret *et al.* 2010; Wood *et al.* 2010; Cocco *et al.* 2010), endometrial (Cocco *et al.* 2010), uterine (Cocco *et al.* 2009), and melanoma (Findeisen *et al.* 2009). These correlative studies were interesting but generally were not introduced as clinical markers. More recently, proteomics has substantially enlarged the scope of reports.

Finding local SAA production within tumor tissues added a new dimension to biologic relationship(s). Colorectal (Gutfeld *et al.* 2006), ovarian (Urieli-Shoval *et al.* 2010) and uterine (Cocco *et al.* 2009, 2010) tumor tissues showed SAA production. Murine SAA3 is prominently expressed in cancer-associated fibroblasts in pancreatic ductal adenocarcinoma (Djurec *et al.* 2018) where, in addition to SAA detection as a biomarker, the cells themselves could stimulate tumor cell growth. Prominent SAA expression on melanoma cells correlated with immunosuppressive neutrophils (surface markers CD11b+CD15+) within the tumor as well as IL-10 (De Santo *et al.* 2010). Thus, tumor (or contiguous stromal) production of SAA may be directly related to downregulation of antitumor immunity.

Further evidence linking SAA and tumor behavior has been shown in several systems. Hansen *et al.* (2015) related SAA 1 (and A3) in mice and SAA1 in humans to the S100 protein S100A4, a marker of metastasis and inflammation. SAA 1 and 3 and transcriptional targets of S100A4 in mice via at TLR4/NF-κB pathway. Their data establish SAA as “effectors for the metastasis-promoting functions of S100A4.”

Knebel *et al.* (2017) found that SAA1 was both expressed and secreted by human glioblastoma cells. SAA production was associated with tumor associated macrophages of the M2 type. Such an association would be consistent with a role in promoting angiogenesis and metastasis. Thus, SAA could have a feedback role in reducing anti-tumor effects of macrophages by inducing transition to the M2 type of functions.

While using SAA as a biomarker may have clinical utility as a “biomarker”, a larger question relates to direct SAA participation in tumor biology. As noted above, SAA interaction(s) as well as the function(s) and phenotype(s) of local macrophages could be central to this question and directly related to understanding tumor immunity. This extremely important and complex topic is beyond the scope of this current review.

## SAA and tissue remodeling - matrix metalloproteinases and collagenase

Tissue remodeling in response to inflammation, trauma or other damage is, at least in part, mediated by enzymatic degradation of proteins in the extracellular matrix (ECM). Matrix metalloproteinases (MMP) are central to this process and the neutral proteinase collagenase has been examined closely. In a series of studies, Brinckerhoff *et al.* evaluated collagenase mRNA levels in rabbit synovial fibroblasts in response to inducers including phorbol myristate acetate (PMA) and monosodium urate (Brinckerhoff *et al.* 1982). Collagenase production was regulated by both inhibitory and stimulatory proteins released by these cells (Brinckerhoff *et al.* 1985; Brinckerhoff and Mitchell 1988). N-terminal sequencing of the secreted proteins identified β_2-_microglobulin and a protein with homology to SAA (Brinckerhoff *et al.* 1989). Adding either of these proteins to synovial cells led to collagenase release, consistent with an autocrine pathway that could both provoke and prolong inflammatory remodeling. The “SAA-like” protein sequence they identified differed from SAA1/2 but was quite similar to murine SAA3 (as well as human SAA3 - originally designated as GSAA1 – (Larson *et al.* 2003a)) and, as will be noted below in more detail, this SAA species uniquely contains the TFLK aa sequence near its N-terminus. Antibody raised against this protein could abolish this induction (Mitchell *et al.* 1991).

Collagenase and stromelysin both could degrade rabbit SAA3 and human SAA, consistent with a feedback mechanism controlling autocrine (or paracrine) effects (Mitchell *et al.* 1993). SAA cleavage occurred in the aa 50-57 region (conserved between both species) leaving an identifiable N-terminal fragment. (C-terminal residues were not identified and may have been degraded further.) Further studies confirmed that MMPs could cleave SAA (and SAA-amyloid fibril proteins) in the same aa 50-57 region and also identified other cleavage sites toward the N-terminus (Stix *et al.* 2001). In the rabbit, SAA3 functioned as an intermediate in the IL-1α stimulation pathway (Strissel *et al.* 1997). SAA also stimulated release of MMPs from human synovial fibroblasts (Migita *et al.* 1998). IL-1 and IL-6 induction of SAA synthesis and release in the same cells required both factors Sp1 and SAF (see also above) acting on promoter elements between nt -254 and -226 (Ilay *et al.* 1999).

Vallon *et al.* (2001) detected local SAA gene expression in rheumatoid arthritis tissues and showed that it induced MMP transcription. Synovial tissue from patients with inflammatory arthritis showed local expression of both SAA and formyl peptide receptor-like (FPLR1) genes as well as MMP production (O'Hara *et al.* 2004). SAA transcription also was found in inflammatory synovium from caprine retroviral arthritis by *in situ* hybridization (Sack and Zink 1992). Mullan *et al.* (2006) using synoviocytes from individuals with rheumatoid arthritis as well as human microvascular endothelial cells found that SAA induced expression of intracellular adhesion molecule (ICAM-1), vascular cell adhesion molecule 1 (VCAM-1) and MMP 1. SAA induced IκBα degradation and NF-κB translation in these cells, implicating this pathway. These observations are consistent with a role for SAA in prolonging local inflammation and tissue degradation by inducing at least one component of the tissue remodeling pathway in both rheumatologic and atherosclerotic tissues. It is important to note that all of these studies did not specifically identify the SAA species (or sequence) used although detailed review shows that SAA1 and 2 were likely to be the species involved; later studies indicate that this may be important. Although the C-terminal regions of most SAA family members are quite similar (and likely cross-hybridize – see above) the N-terminal regions have multiple differences, including the characteristic TFLK sequence near the N-terminal end (see below).

Rosiglitazone (a thiazolidinedione) decreased MMP-9, SAA and TNF levels, consistent with common control site(s) for inflammation in individuals with diabetes (Marx *et al.* 2003; Ghanim *et al.* 2006). Human synovial fibroblasts contained mRNA for both SAA and formyl peptide receptor-like-1 (Dufton *et al.* 2010; Marsche *et al.* 2007) and exogenous SAA could induce MMP-1 and 3 in these cells. In mice transgenic for SAF-1, infection with *Borelia burgdorferi* led to inflammatory arthritis as well as MMP-1 induction (Ray *et al.* 2004b). In human THP1 cells SAA induced MMP9 synthesis through FPRL1 (Lee *et al.* 2005). MMP9 transcription responds to SAF-1 and AP1 whose binding sites are separated by only 14 nt in the upstream control region (Ray *et al.* 2005). SAA 1.1 was more susceptible to degradation by MMP1 than SAA1.5, correlating with a polymorphism at aa 57 (van der Hilst *et al.* 2008).

## SAA and pulmonary physiology and disease - sarcoidosis

Lung changes in chronic obstructive pulmonary disease (COPD) are associated with inflammation and tissue destruction. Serologic markers correlating with acute exacerbations have been sought - CRP levels are related to functional decline in affected patients (Donaldson *et al.* 2005; Man *et al.* 2006).

Using SELDI-ToF proteomics, Bozinovski *et al.* (2008) identified SAA in sera of individuals with acute, glucocorticoid-refractory exacerbations of COPD and quantified the changes by ELISA. SAA levels were better correlated with clinical status than those of CRP. Subsequently, they studied bronchoalveolar lavage fluid (BALF) where there was good correlation between levels of SAA, IL-8 and neutrophil elastase (Bozinovski *et al.* 2012). Their data implicated alveolar macrophages as a potential source of SAA in BALF. There was no evidence for AA fibril deposition in affected tissues. Using human lung epithelial cells with and without the FPRL1 receptor showed that the receptor was required in order for SAA to induce significant amounts of the monocyte chemoattractant protein-1 (MCP-1), GMN-CSF and IL-8. This effect was antagonized by LXA4 and 15-epi-LXA4. Interestingly, despite their recognized effects in reducing other inflammatory reactions, glucocorticosteroids increased SAA production. Glucocorticosteroids can increase FPRL1 expression in leukocytes (Sawmynaden and Perretti 2006) and, as noted above, SAA1 has a glucocorticoid-responsive element (Thorn and Whitehead 2002, 2003). These observations were consistent with the lack of general efficacy of glucocorticosteroids in treating COPD exacerbations. They also revealed potential antagonism for LXA4 derivatives.

SAA has been implicated in the pathophysiology of sarcoidosis, a chronic, systemic, inflammatory disorder characterized by formation of noncaseating granulomata (Chen and Moller 2010). Local production of TH-1 cytokines and TNF have been recognized. Using a proteomics-based approach, Moller *et al*. (Song *et al.* 2005; Chen *et al.* 2010) identified IgG responses to the catalase-peroxidase protein from *M. tuberculosis* (mKatG) in >50% of affected individuals. BALF from sarcoidosis patients showed significantly higher levels of SAA. Tissue staining localized SAA to macrophages and multinucleated giant cells and was compatible with the notion that SAA is regulated by local CD3 T cells. SAA was prominent in areas of fibrosis. Studying the human macrophage-like cell line THP-1 they showed SAA stimulation of NF-κB that could be inhibited by preincubation with anti-TLR2 antibodies. A competitive binding ligand (Pam_3_CSK_4_) had a similar effect. They noted that polymorphisms in both RAGE and TLR2 have been associated with sarcoidosis. Interaction of SAA with multiple receptors (as recounted above and Table 1) is consistent with involvement of multiple pathways in regulating the granulomatous changes typical of sarcoidosis. They proposed that SAA is central to a specific (possibly unique) pathway for “epithelioid granulomatous inflammation in association with polarized Th1 responses to specific mycobacterial antigens.”

## Cytokine-like function, intestinal physiology

Host defense against intestinal pathogens has revealed an important SAA role. Ivanov *et al.* (2009) reported specific, IL-17-secreting, Th17 cell accumulation in the lamina propria of the murine intestine as a specific consequence of non-invasive segmental filamentous bacteria (sfb) adhesion. Sfb adhesion to the intestinal epithelial cells (not simply colonization) led to SAA1 transcription as the most prominent upregulated species. Adding SAA to cocultures of dendritic cells and naïve CD4^+^T cells led to a concentration-dependent induction of Th17 cell differentiation (including cytokines and RORγt).

Atarashi *et al.* (2015) proposed that actin reorganization in the epithelial cells secondary to sfb adhesion led to elevated C/EBPδ expression which they proposed interacted with 2 DNase hypersensitive sites 3’ of the SAA1 gene. C/EBPδ levels were upregulated in sfb-colonized mice where they emphasized the need for direct contact between the cells and the sfb.

Sano *et al.* (2015) emphasized that IL17A production was greatest in the ileum. They identified type 3 innate lymphoid cells (ILC3) which, following sfb adhesion, secreted IL22. This response then led to SAA production by the epithelial cells through a STAT-dependent mechanism. The SAA effect on Th17 cells appeared to resemble cytokine activation. Binding of retinol derivatives to SAA (as suggested by Derebe *et al.* (2014)) could provide another role for SAA in maintaining intestinal barrier integrity. Pedicord and Mucida (2015) emphasized that these studies implicate “local environmental cues in licensing effector cytokine production.” Gury-BenAri *et al.* (2015) distinguished transcriptional differences between antigen-presenting cells that inhibit microbiota-directed T cell responses from those that produce IL11 and promote T cell responses (although SAA transcription was not reported). Mao *et al.* (2018) emphasized the role of ILC3 cells in maturation of gut commensalism.

Studying mice, Reigstad *et al.* (2009) showed that gut bacteria and possibly other bacterial products were associated with SAA3 expression in both adipose and colonic tissue. The Toll-like receptor/MyD88/NF-κB signal was at least partially responsible for the colon response. Gardiner *et al.* (2009) showed that using 10 or 42 aa N-terminal polypeptides from SAA3 *in vitro* to pretreat HT29 cells reduced adherence of enteropathogenic *E. coli* by 72%. However, using either polypeptide to pretreat mice did not reduce Salmonella invasion, consistent with likely participation of other components *in vivo*.

## Maternal/fetal health

### Preterm labor and sepsis

Serum SAA levels have been evaluated as markers for preterm labor. In mice, using a model of intrauterine LPS injection, Yang *et al.* (2009) found that SAA2 levels were increased in animals with preterm delivery. In women with unexplained recurrent early pregnancy loss Ibrahim *et al.* (2017) found significant elevations of SAA level (p<0.001). They identified SAA as “an independent indicator of primary unexplained recurrent early pregnancy loss after adjusting for maternal age and gestational age.” Sandri *et al.* (2014) showed that SAA at moderate levels enhances placentation via a TLR-4 receptor and that increasing SAA levels can disturb it. Mithal *et al.* (2017) found that elevated cord blood levels of SAA, CRP and haptoglobin levels were each associated with early onset neonatal sepsis in preterm (29.7 weeks) infants.

## Mammary-derived SAA

McDonald *et al.* found SAA-reactivity in colostrum from cow, horse and sheep (McDonald *et al.* 2001) using a monoclonal antibody ELISA (McDonald *et al.* 1991). Using both batch purification and affinity chromatography they isolated substantial quantities of the protein (relatively little was found in mature milk) and sequencing revealed a sequence corresponding to the sequence reported earlier in rabbit (Brinckerhoff *et al.* 1989) as well as that predicted for human SAA 3 (Sack and Talbot 1989). The N-terminal region of this protein differed from that for the serum protein and there was no elevation in serum SAA levels in the animals. All isolates contained a 4 aa TFLK sequence near the N-terminus.

Adding either a 10 aa polypeptide containing the TFLK sequence or the 4 aa TFLK peptide alone to HT29 human intestinal epithelial cells led to intestinal MUC3 production (Larson *et al.* 2003b). A polypeptide corresponding to the human SAA3 gene sequence (GWLTFLKAAG) produced the same effect and led to a 73% decrease in adherence of enteropathogenic *E. coli* to the cultured cells (Mack *et al.* 2003) (later confirmed by Gardiner *et al.* (2009) as noted above).

Prolactin or LPS stimulation of a bovine mammary cell line (CLR-10274) revealed only SAA3 expression by RT-PCR (other SAA species were not transcribed) implying tissue-specific control (Larson *et al.* 2005a). Adding lipoteichoic acid from *S. aureus* to bovine mammary gland cells confirmed stimulation of SAA synthesis consistent with the potential use of SAA3 as a biomarker for mastitis (Weber *et al.* 2005). Detailed analysis of the bovine SAA3 gene structure identified an enhancer region between nt -2571 and -2338 but also a silencer region between nt -2338 and -1003. The minimal fragment retaining responsiveness was 352 nt and 53 nt of the untranslated region of exon 1 also enhanced expression (Larson *et al.* 2005b). LPS remained the most effective stimulant.

As discussed earlier, following LPS or prolactin stimulation, human mammary gland epithelial cell lines MCF-7 and T47-D showed SAA3 transcripts and RACE revealed a 655 bp cDNA sequence including an open reading frame corresponding to a 42 aa protein with truncation in the C-terminal region due an early stop codon. The corresponding short protein was not detected ((Larson *et al.* 2003a), see above). SAA3 was not found in human colostrum. An earlier report suggested that SAA3 was present (based on ELISA activity alone (Knee *et al.* 2015)). However, further study showed that human colostrum contains only a full-length, 12 kD protein shown by MS/tof sequencing to be SAA1.1; no other reactive species were detected (Sack *et al.* 2018). Finding SAA1.1 in human colostrum implies evolutionary pressure to change expression of at least SAA1.1 in human mammary cells. SAA1.1 does not contain the TFLK aa sequence (found in mice and other mammals *e.g*. (McDonald *et al.* 2001)) and whether it also contributes to intestinal mucin secretion is not yet known. Nevertheless, finding this switch in expression to another gene family member is consistent with possible evolutionary selection for a molecule with a likely important role in host defense.

## Conclusions

Since its discovery, SAA has been an enigma. Nevertheless, its impressive evolutionary conservation as well as its dynamic synthesis control have always implied a consequential position in biology. SAA’s participation in the primordial survival physiology of the APR is consistent with both the evolutionary preservation and stability of SAA genes and proteins and with their cytokine and chemokine relationships (see Fig. 7). SAA helps link the complex network of cells (including macrophages, hepatocytes, MDSC) and proteins mediating inflammation.

In humans, clinical aspects of SAA protein pathobiology largely present later in life and thus may be appear to be “side effects” of generic APR preservation. For example, the 3-dimensional and amphipathic features of the SAA monomer help explain complex lipid interactions including HDL formation and cholesterol transport (details unresolved). Combining lipid associations with inflammatory cells and proteins is central to development of atherosclerosis. Stimulation (possibly via feedback loop(s)) of proteinase secretion contributes to chronic tissue injury in arthritis as well as pulmonary damage in sarcoidosis. Mediating signals reflecting the intestinal bacterial environment can modulate host immune defense(s) and possibly the gut flora itself. SAA’s presence in colostrum, stimulating neonatal intestinal mucin production, is consistent with SAA’s preservation as a “pro-survival” molecule. Other aspects of maternal-fetal health are characterized by SAA’s value as a biomarker for both prematurity and mastitis. Not surprisingly, myriad gene and cell surface changes can implicate SAA as a biomarker in malignancy. However, likely even more important is the relationship of SAA to immunologic pathways involving macrophage biology that are related to both metastasis and treatment including immunotherapy.

Finally, the propensity of fragment(s) of SAA monomers to form highly ordered amyloid fibrils explains many pathophysiologic features of “secondary” amyloidosis where chronic inflammation (or periodic – *e.g.* familial Mediterranean fever) overwhelms degradation that otherwise would occur under baseline conditions. It is ironic that “Serum Amyloid A” - the name acquired for this molecule soon after its description - (and now applied to the entire gene and protein family) is based on this property which now appears to be an epiphenomenon based on its unique protein structure and inherent propensity to form fibrils rather than its basic, well preserved physiologic function(s).

Convergence of multiple disciplines has begun to clarify the multifaceted relationships of SAA proteins and helps explain their remarkable evolutionary preservation. In addition to many valuable descriptive aspects of SAA biology, possibilities for prevention and specific, molecular-based treatments are emerging.

## Abbreviations

ABCA: ATP-binding cassette (transporter)
ACAT: Acyl-CoA:cholesterol acyl transferase
APR: Acute phase response
BALF: Broncho-alveolar lavage fluid
C/EBP: CCAAT/enhancer-binding protein
CD: Circular dichroism
CEH: Cholesterol ester hydrolase
CHO: Chinese hamster cell (line)
COPD: chronic obstructive pulmonary disease
CRP: C-reactive protein
DNA: Deoxyribonucleic acid
ELISA: Enzyme-linked immunosorbent assay
fMLP: Formylmethionine-leucyl-phenylalanine
FPRL: Formyl peptide receptor-like
gp130: Glycoprotein130 – class of cytokine receptor
HDL: High-density lipoprotein
HepG2: Cell line
ICAM: Intercellular adhesion molecule
IFN-: Interferon-
IL-: Interleukin-
Kb: Kilobase
LCAT: Lecithin/cholesterol acyl transferase
LPS: Lipopolysaccharide
MAPK: Mitogen-activated protein kinase
MDSC: Myeloid-derived suppressor cell
MMP: Matrix metalloproteinase
mRNA: Messenger RNA
NMR: Nuclear magnetic resonance
PAMPS: Pathogen-associated molecular patterns
PMA: Phorbol myristic acid
PMN: Polymorphonuclear leukocyte
RACE: Rapid amplification of cDNA ends (technique)
RAGE: Advanced glycation end product receptor
RNA: Ribonucleic acid
RT-PCR: Reverse transcriptase-polymerase chain reaction
SAA: serum amyloid A
SAF: SAA activating factor
SAP: Serum amyloid P
sfp: Segmented filamentous bacteria
SNP: Single nucleotide polymorphism
SR-B1: Scavenger receptor-= B1
TLR: Toll-like receptor
TNF: Tumor necrosis factor
VCAM: Vascular cell adhesion molecule
